# Photocatalytic Abstraction of Hydrogen Atoms from Water Using Hydroxylated Graphitic Carbon Nitride for Hydrogenative Coupling Reactions

**DOI:** 10.1002/anie.202204256

**Published:** 2022-04-11

**Authors:** Dongsheng Zhang, Pengju Ren, Wuwen Liu, Yaru Li, Sofia Salli, Feiyu Han, Wei Qiao, Yu Liu, Yingzhu Fan, Yi Cui, Yanbin Shen, Emma Richards, Xiaodong Wen, Mark H. Rummeli, Yongwang Li, Flemming Besenbacher, Hans Niemantsverdriet, Tingbin Lim, Ren Su

**Affiliations:** ^1^ Soochow Institute for Energy and Materials InnovationS (SIEMIS) Soochow University Suzhou 215006 China; ^2^ SynCat@Beijing, Synfuels China Technology Co. Ltd. Leyuan South Street II, No.1, Yanqi Economic Development Zone C#, Huairou District Beijing 101407 China; ^3^ State Key Laboratory of Coal Conversion Institute of Coal Chemistry Taiyuan 030001 China; ^4^ School of Chemistry Cardiff University Park Place Cardiff CF10 3AT UK; ^5^ Suzhou Institute of Nano-Tech and Nano-Bionics (SINANO) No. 398 Ruoshui Road, Suzhou Industrial Park Suzhou 215123 China; ^6^ Interdisciplinary Nanoscience Centre (iNANO) Aarhus University Gustav Wieds Vej 14 DK-8000 Aarhus C Denmark; ^7^ SynCat@DIFFER Syngaschem BV 6336 HH Eindhoven The Netherlands; ^8^ Joint School of National University of Singapore and Tianjin University International Campus of Tianjin University Bin-hai New City Fuzhou 350207 China

**Keywords:** Coupling Reaction, Heterogeneous Photocatalysis, Hydrogen Atom Transfer, Surface Hydroxylation, Water

## Abstract

Employing pure water, the ultimate green source of hydrogen donor to initiate chemical reactions that involve a hydrogen atom transfer (HAT) step is fascinating but challenging due to its large H−O bond dissociation energy (BDE_H‐O_=5.1 eV). Many approaches have been explored to stimulate water for hydrogenative reactions, but the efficiency and productivity still require significant enhancement. Here, we show that the surface hydroxylated graphitic carbon nitride (gCN−OH) only requires 2.25 eV to activate H−O bonds in water, enabling abstraction of hydrogen atoms via dehydrogenation of pure water into hydrogen peroxide under visible light irradiation. The gCN−OH presents a stable catalytic performance for hydrogenative N−N coupling, pinacol‐type coupling and dehalogenative C−C coupling, all with high yield and efficiency, even under solar radiation, featuring extensive impacts in using renewable energy for a cleaner process in dye, electronic, and pharmaceutical industries.

## Graphical abstract

Using water as hydrogen donor is very challenging for hydrogen atom transfer reaction. Compared with gCN, the surface hydroxylated graphitic carbon nitride (gCN−OH) shows the activation of H−O bonds in water under visible light irradiation, thus rendering water as hydrogen donor for multiple reductions involving a hydrogen atom transfer (HAT) step and the oxidation of water to H_2_O_2_.

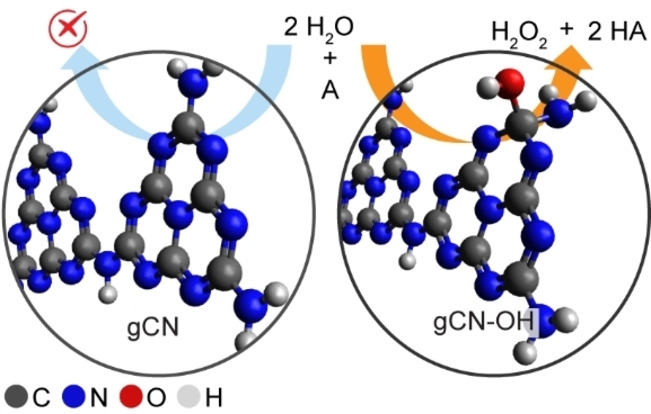

## Introduction

Chemical reactions involving a hydrogen atom transfer (HAT) step are widely employed in natural synthesis and catalytic reactions in the chemical industries.^[1]^ Molecular hydrogen, alcohols and metal hydrides are obviously the most often used hydrogen donors for a variety of reactions.^[2]^ Employing water as the hydrogen donor is a low cost and CO_2_ emission‐benign process that shows huge economic and environmental potential. As one type of proton‐coupled electron transfer (PCET) reaction,^[3]^ a direct HAT process does not require harsh reaction conditions nor noble metal‐based catalysts, however, activation of water under mild conditions is a grand challenge due to its high thermostability.

It has been observed that micro‐sized water droplets become reactive for the hydrogenation of C=C, C=O and S−S bonds at trace levels (≈nM) in the absence of a catalyst.^[4]^ The confinement of water into nanodomains can strongly enhance the electrochemical water reduction.^[5]^ Catalytic routes further facilitate the activation of water for reactions. The hydrogenation of a C=C bond in unsaturated carbonyl compounds is achieved using a phosphetane oxide catalyst with a robust organosilane reductant.^[6]^ Electrocatalytic water oxidation provides an alternative pathway for selective hydrogenation of acetylene,^[7]^ nitrobenzene,^[8]^ and aldehydes,^[9]^ although designed electrocatalysts and precious ion exchange membranes are required. Photocatalytic hydrogenation of ketones, alkenes, and nitroaromatics can be realized under proper irradiation in the presence of electron donors (e.g., Na_2_SO_3_, alcohols, triethylamine),^[10]^ which are oxidized instead of water; moreover, in the case of organic electron/hydrogen donors, the hydrogen source is unlikely to be water.^[11]^ A recent work shows that photocatalytic hydrogenation of CO_2_ using pure water is feasible, though the yield (0.8 μmol) and reaction rate (≈0.15 μmol h^−1^) needs significant enhancement for large‐scale applications.^[12]^

**Scheme 1:**
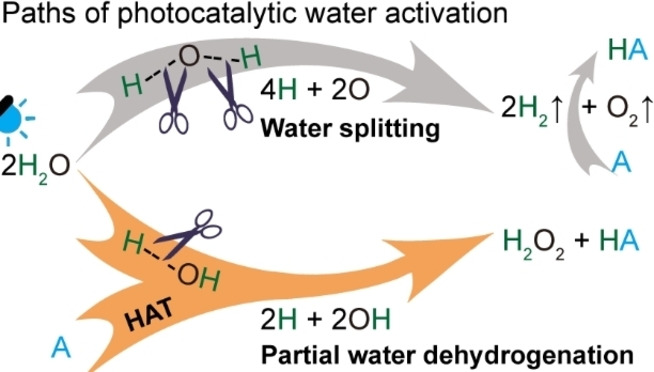
Paths of photocatalytic water activation for HAT reactions. Top: water splitting path; Bottom: partial water dehydrogenation path.

Performing HAT reactions using the molecular hydrogen produced from photocatalytic water splitting presents a simple strategy that potentially allows the utilization of solar energy (Scheme 1, top). Though only 1.23 eV is needed to split water thermodynamically, the sluggish kinetics suggest that both efficient photocatalysts for water splitting and a robust catalyst for the step‐wise hydrogenation reactions are required, thus resulting in complicated catalyst design and low efficiency. Additionally, removal of the liberated O_2_ from the gaseous mixture is required. In comparison, direct hydrogenation of the electron acceptor (A) using hydrogen atoms that are produced from partial dehydrogenation of water via dissociation of the H−O bond saves the expenses for gas separation and H_2_ dissociation (Scheme 1, bottom). Previous investigations confirm that hydrogen peroxide can be produced via the partial reduction of oxygen or dehydrogenation of water,^[13]^ yet the utilization of abstracted hydrogen from water is ignored and rarely reported, making it less atom efficient. However, one should be aware that this route is thermodynamically challenging due to the high bond dissociation energy (BDE) of the H−O bond in water (BDE_H‐O_=5.1 eV),^[14]^ thus a designed photocatalyst is needed to reduce the BDE_H‐O_ into a level that can be initiated by visible light (<3 eV).

Recent works show that graphitic carbon nitride (gCN) based photocatalysts are capable of performing a series of photocatalytic HAT reactions using alcohol as the hydrogen donor under visible light irradiation. The catalytic performance in terms of kinetics and selectivity can be further tuned by precise engineering of the supported metal cocatalyst on gCN.^[15]^ Alternatively, the gCN photocatalyst is also a decent platform for microstructural and surface active sites engineering to boost mass diffusion and extraction of hydrogen.^[16]^ In addition, band gap engineering of gCN is also feasible to further enhance light absorption and photocatalytic activity.^[17]^ This implies that a purposely designed gCN photocatalyst may achieve HAT reactions using water as the hydrogen donor under visible light radiation.

Here, we have designed a hydroxylated graphitic carbon nitride (gCN−OH) photocatalyst to promote the abstraction of hydrogen atom from pure water in the absence of electron donors (scavengers). The gCN−OH photocatalyst enables partial dehydrogenation of water into H_2_O_2_ under visible light irradiation, allowing the utilization of H atoms for a series of reductive coupling reactions that involve HAT steps. The mechanism of surface hydroxylation towards water dissociation and its practical applications are discussed.

## Results and Discussion

**Figure 1:**
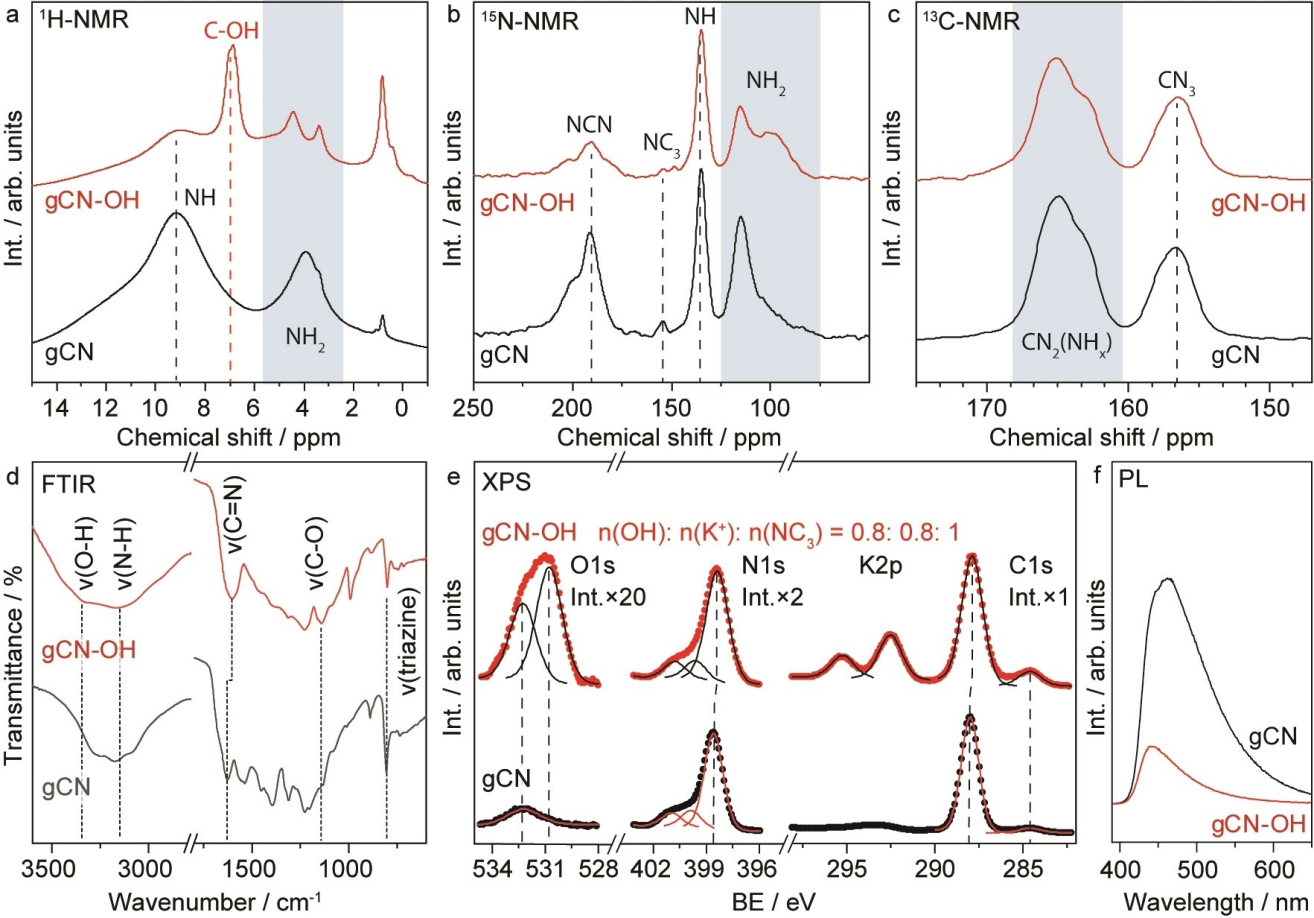
Structural characterization of the photocatalyst. a)–c) ^1^H, ^15^N and ^13^C ssNMR of pristine gCN synthesized from thermal pyrolysis of ^15^N labelled urea and gCN−OH from KOH treatment of the as‐synthesized ^15^N labeled gCN. d)–f) FTIR, XPS and PL of gCN and gCN−OH.

The gCN−OH is synthesized employing an anti‐solvent method by dosing aqueous KOH solution (4 M) into a 1,4‐dioxane‐gCN suspension (Note S1). This optimized procedure allows concentrated KOH to etch the pristine gCN more efficiently, thus providing a stable hydroxylated surface with dense hydroxyl groups compared with reported methods that use H_2_O_2_ as the etching agent.^[17]^ Solid state nuclear magnetic resonance (ssNMR) confirms the successful hydroxylation of gCN. The new peak at 7 ppm in the ^1^H spectra of gCN−OH can be attributed to hydroxyls (Figure 1a). Density functional theoretical (DFT) calculations indicate that the OH is attached to the carbon atom neighboring an amino group of the melem unit (Figure S1). The ^15^N and ^13^C spectra reveal that the melem skeleton remains after surface hydroxylation (Figures 1b and c). The appearance of a shoulder on the peak at 100 ppm observed in the ^15^N spectra can be assigned to the amino group adjacent to the C−OH group, in agreement with the structural assignment predicted via DFT. Attenuated total reflection infrared spectroscopy (ATR‐IR) analysis reveals that the triazine structure in gCN−OH is preserved according to the vibrational peak at 810 cm^−1^ (Figure 1d).^[18]^ An obvious broadening of the peak at ≈3200 cm^−1^ is observed for the gCN−OH in comparison with the pristine gCN, which can be linked to the OH stretching of the surface hydroxyls (3340 cm^−1^). A slightly red‐shifted *ν*(C=N) compared to that of pristine gCN indicates that the C=N stretching in gCN−OH is caused by the neighboring OH functionalization of the C atom. An additional vibrational peak at ≈1140 cm^−1^ observed for gCN−OH can be assigned to the formation of C−O bonds.^[18]^ An evolved peak at 990 cm^−1^ is also observed for the gCN−OH, which is most likely associated with the combination of v(C−O) and v(C−N) of the triazine skeleton according to our calculations (Table S1).

X‐ray photoelectron spectroscopy (XPS) also confirms the presence of surface oxygen species in gCN−OH in the form of hydroxyl (532.3 eV, Figure 1e, O 1s). Meanwhile, the N1s and C1s spectra of the gCN−OH remain unchanged compared to those of gCN (Figure 1e). The estimated atomic ratio of OH : NC_3_ : K^+^ is ≈0.8 : 1 : 0.8 according to the XPS survey scan (Figure S2), confirming that each melem unit holds ≈1 OH (equivalent to 0.5 wt % of OH, Note S2). Note that incorporation of K^+^ in the layered structure enhances the stability of gCN−OH according to previous studies.^[19]^ The intensity of the O 1s peak remains unchanged after repeated washing, confirming that the surface OH functional groups form a chemical bond with the carbon atoms within the gCN−OH (Figure S3). In addition, a shoulder peak at ≈287.2 eV of the C K‐edge spectra observed for gCN−OH further confirms that the surface hydroxyls are indeed bound to carbon atoms according to the X‐ray absorption near edge spectroscopy (XANES, Figure S4).^[20]^ Noticeably, the presence of surface hydroxyls in gCN−OH strongly suppresses the recombination of the photogenerated charge carriers, which also benefits photocatalytic reactions, as evidenced by the photoluminescence spectra (PL, Figure 1f). The as‐synthesized gCN−OH presents a slightly enhanced light absorption, but has otherwise similar properties in terms of optical band gap, crystallographic structure and microstructures compared to pristine gCN (Figure S5).

**Figure 2:**
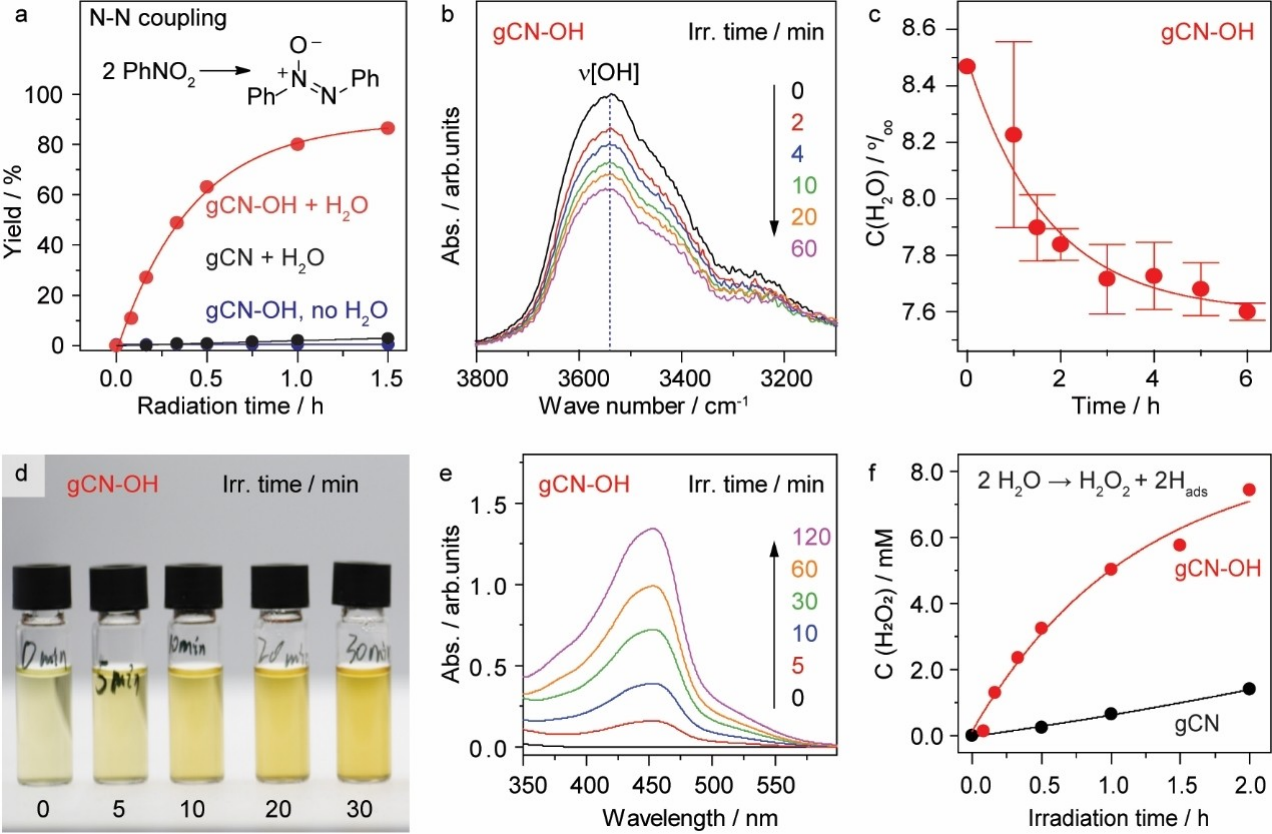
Photocatalytic performance. a) Time course of nitrobenzene reductive homocoupling for the synthesis of azoxybenzene using gCN−OH and gCN. Reaction conditions: 10 mg photocatalyst in 2 ml 1 vol % water–dioxane solution with 8 mM reactant and 40 mM KOH under 410 nm irradiation (30 mW cm^−2^) and 1 bar N_2_ at room temperature (RT). b), c) Consumption of water during photocatalytic nitrobenzene reduction using gCN−OH determined by DRIFT and MS, respectively. d)–f) Titration of evolved H_2_O_2_ during photocatalytic reductive coupling of nitrobenzene. d) Images of the centrifuged gCN−OH reaction suspensions after dosing CuSO_4_ and DMP. e) UV/Vis spectra of the titrated solution. f) Evolution of H_2_O_2_ using gCN and gCN−OH.

We have first examined the gCN−OH photocatalyst for reductive N−N coupling of nitrobenzene in a 1 vol % water‐dioxane solution under visible light irradiation (Figure 2, Figures S6 and S7, and Note S3). The value‐added homo‐coupling compounds are more favored products than the fully hydrogenated compounds (aniline) due to the formation of active nitrosobenzene and N‐phenylhydroxylamine intermediates according to previous study.^[11]^ These intermediates are detectable by mass spectrometry, confirming that hydrogen is indeed involved in the reaction (Figure S8). Obviously, the presence of gCN−OH photocatalyst and water is essential for the formation of azoxybenzene via reductive N−N coupling of nitrobenzene with 1 vol % of water (Figure 2a, Table S2). Here, the abstracted hydrogen atoms from water first reduce nitrobenzene to nitrosobenzene and N‐phenylhydroxylamine (NPH) radicals, which then convert into azoxybenzene via dehydrative coupling (≈90 % yield after 1.5 h reaction). No reaction is observed in the absence of water, indicating that the hydrogen originates from water rather than the photocatalyst. Optimum performance is observed for the gCN−OH prepared using 4 M of KOH (Table S3). The pristine gCN exhibits negligible catalytic performance even in the presence of KOH, suggesting the key role of the surface hydroxyl functional group.

The involvement of water in the photocatalytic reactions is studied by spectroscopic analysis. The consumption of water molecules, reflected in a decrease of the OH stretching vibration (ν[OH]=3540 cm^−1^), is observed upon irradiation in the presence of nitrobenzene from the in situ diffuse reflectance infrared Fourier transform spectroscopy (DRIFT, Figure 2b). This confirms the continuous H−O bond dissociation of water in the presence of the gCN−OH photocatalyst. This result is supported by in situ gas chromatograph‐mass spectrometry (GC‐MS), which demonstrates that the consumption of water follows first‐order kinetics during photocatalytic conversion of nitrobenzene to NPH (Figure 2c). Noticeably, mass balance analysis shows that the quantity of consumed water during the reaction (≈0.75 mmol) matches closely the quantity of water required (0.72 mmol) for the reduction of nitrobenzene, if we assume that each water molecule donates one H atom in the reaction, thus confirming that water is the only source of H atoms (Note S4).

The dehydrogenative product of water (most likely H_2_O_2_) is further confirmed by UV/Vis spectrometry of the aliquots of the centrifuged photocatalyst suspension after irradiation.^[21]^ The pale yellow CuSO_4_‐2,9‐dimethyl‐1,10‐phenanthroline (DMP) solution gradually develops into dark yellow upon increasing the irradiation time of the photocatalyst suspension (Figure 2d), indicating that more Cu^2+^ is reduced by the increased concentration of photogenerated H_2_O_2_, as shown in the UV/Vis spectra (Figure 2e). Since the reaction is performed under deaerated conditions, the formation of H_2_O_2_ can only come from water oxidation. Quantitative analysis shows that the evolution of H_2_O_2_ on gCN−OH follows an exponential growth trend with a rate constant of 0.85 h^−1^ (Figure 2f and Figure S9). The concentration of H_2_O_2_ reaches 8 mM after full conversion of nitrobenzene (8 mM) into azoxybenzene, which is close to the theoretical value of 12 mM in consideration of H_2_O_2_ self‐decomposition (Note S4). In comparison, the gCN photocatalyst produces a negligible amount of H_2_O_2_ under identical conditions, suggesting that the surface hydroxylation indeed promotes the complete redox chemistry under irradiation.

**Figure 3:**
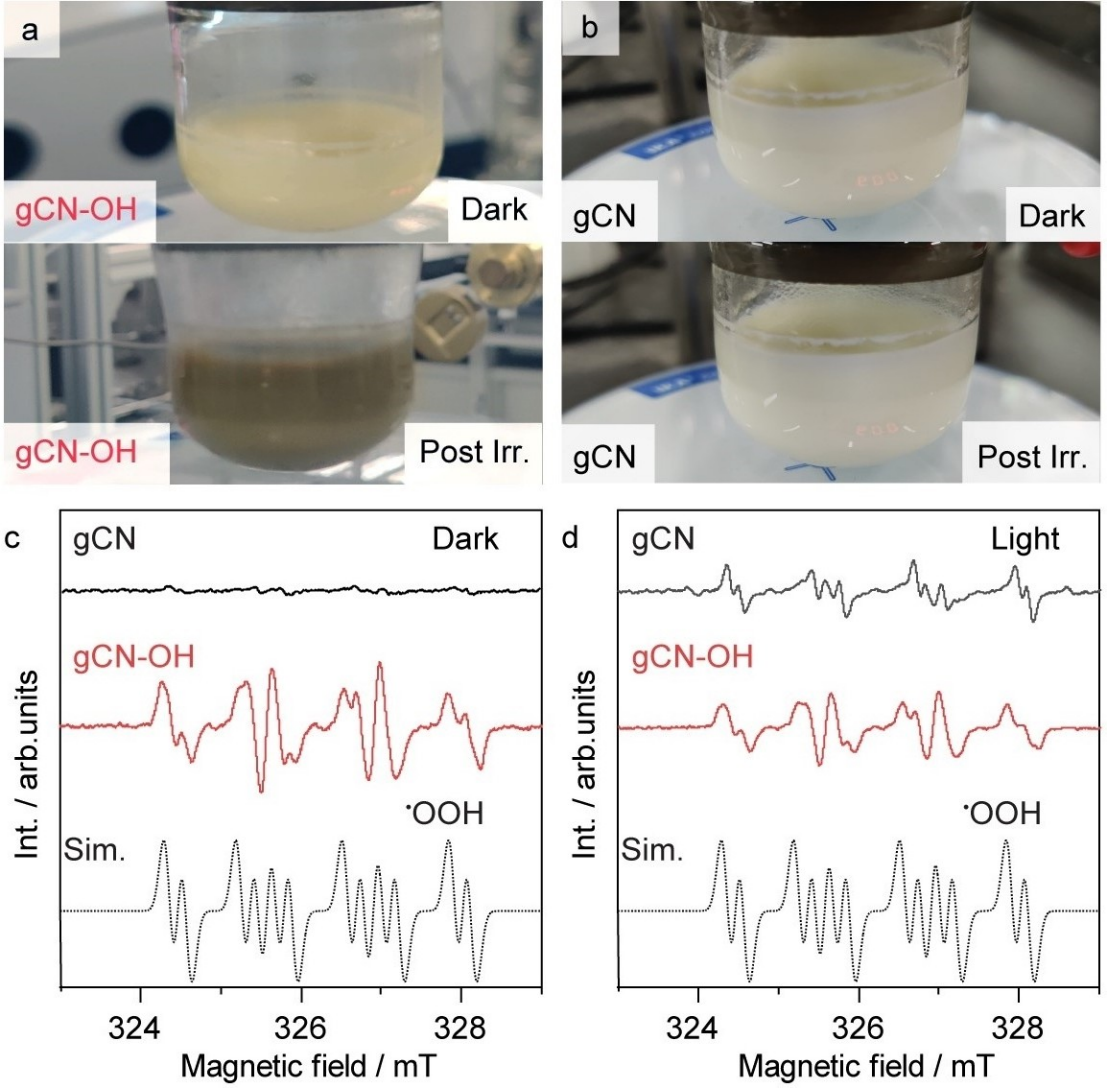
Reaction mechanism analysis. a), b) Images of the fresh gCN−OH and pristine gCN in 1 vol % water‐dioxane under dark and post irradiation under deaerated conditions. c), d) ESR spectra of the photocatalyst‐dioxane suspension with 1 vol % water and spin trap (DMPO). Reaction conditions: 10 mg catalyst in 2 mL 1 vol % water‐dioxane and 8 mM nitrobenzene under deaerated conditions, 410 nm LED (30 mW cm^−2^).

It is noticed that the appearance of the gCN−OH‐solvent suspension changes upon irradiation in the absence of a hydrogen acceptor. While the initially dark yellow gCN−OH suspension turns grey after irradiation (Figure 3a), the pale‐yellow pristine gCN suspension remains unchanged throughout the irradiation (Figure 3b). This change in color corresponds to the formation of H_ads_ on gCN according to our previous investigations,^[11]^ which agrees well with the evolution of H_2_O_2_. To identify the radicals formed during the dissociation of water, electron spin resonance (ESR) is performed employing 5,5‐dimethyl‐1‐pyrroline N‐oxide (DMPO) as the spin trap in 1 vol % water‐dioxane solution (Figure 3c). While the pristine gCN produces a negligible concentration of radical species under dark conditions as indicated by the low intensity spectrum (black line), gCN−OH presents a more intense and complex multiplet signal (red line). The signal presents a characteristic *g*_iso_=2.004, *a*_iso_(^14^N)=37.18 MHz and two distinct proton couplings *a*_iso_(^1^H_β_)=25.2 and *a*_iso_(^1^H_γ_)=5.7 MHz, which can be assigned to the DMPO−⋅OOH adduct.^[22]^ The ⋅OOH radicals most likely form by recombination of ⋅OH radicals on the surface of gCN−OH,^[23]^ which eventually desorb from the catalyst into the solution and are thus available for the formation of an adduct with DMPO.

Upon irradiation, the ⋅OOH signal is observed for the gCN (black line, Figure 3d), indicating that the photogenerated charge carriers on gCN dissociate water into adsorbed ⋅OH that combines into ⋅OOH species. While the intensity of free radicals normally increases upon increasing radiation time due to the accumulation of radicals,^[24]^ the ESR spectrum of the illuminated gCN−OH remains identical to that under dark conditions (red line, Figure 3d). Since the gCN−OH is photo‐active under irradiation, it implies that a large portion of photogenerated oxygen radical species on gCN−OH rapidly converts into stable oxygen species (i.e., H_2_O_2_) that are silent in ESR. This allows the hydrogenated organic intermediates for further coupling reaction by preventing the unwanted back reaction. For pristine gCN, the oxygen radicals rapidly recombine with H_ads_ and turn back to water. In addition, a series of control experiments confirms that the presence of gCN−OH and irradiation are necessary to generate radical species (Figure S10).

**Figure 4:**
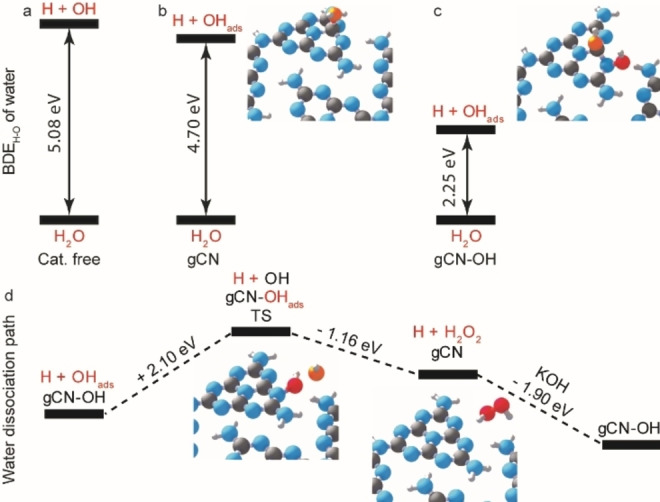
Calculated water dissociation energy and dissociation path on gCN−OH. a)–c) BDE_H‐O_ of water in gas phase, on pristine gCN, and on gCN−OH. d) Reaction coordinates of water dissociation into free hydrogen atom and H_2_O_2_ on gCN−OH. Grey: C, blue: N, white: H, red: O.

The high efficiency of gCN−OH in utilizing water for reactions is investigated by theoretical calculations (Figure 4). While the calculated catalyst‐free dissociation of water into H atom and ⋅OH requires 5.08 eV that matches well with experimental value (Figure 4a),^[25]^ the presence of pristine gCN only slightly reduces the BDE_H‐O_ of water to 4.70 eV (Figure 4b). This results in the formation of a free hydrogen atom and a surface adsorbed hydroxyl (OH_ads_), which preferentially attaches to the N_3_C carbon atom (Figures S11 and S12). The hydroxylated carbon nitride, gCN−OH, is thermally stable (Δ*H*=−1.33 eV), which is in good agreement with ssNMR, ATR, and XPS analysis. Note that the BDE_H‐O_ of water into H and OH_ads_ on gCN−OH is greatly reduced to 2.25 eV (Figure 4c), resulting in the formation of the second OH_ads_ located on the neighboring N atom (NCN) adjacent to the initial OH_ads_ site. This suggests that the presence of gCN−OH can initiate water dissociation upon visible light irradiation (*λ*<550 nm). The BDE_H‐O_ of water on gCN−OH is lower than on gCN because hydroxylating the melem substrate in gCN−OH creates less disruption in the resonance structure as compared to gCN. Furthermore, no significant energy barrier is observed throughout the catalytic cycle of water dissociation on gCN−OH (Figure 4d). The OH_ads_, together with the OH from the gCN−OH, requires only a relatively low activation energy (2.10 eV) to pass the transition state (TS) to desorb and recombine into H_2_O_2_ and yield pristine gCN, which can be easily hydroxylated back to gCN−OH by KOH to complete the catalytic cycle. Our calculations therefore reveal that the utilization of water as a hydrogen donor will be a realistic route if the gCN photocatalyst is initially hydroxylated to avoid the primary dissociation of the first water molecule.

**Figure 5:**
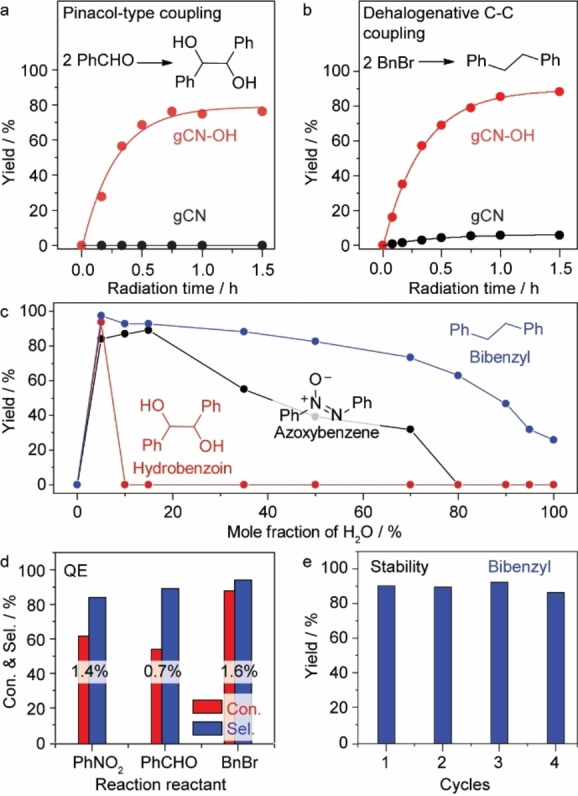
Expandable applications. a, b) Time course of benzaldehyde and benzyl bromide reductive homocoupling for the synthesis of hydrobenzoin and bibenzyl using gCN−OH and gCN in 1 vol % water–DMF solution. c) Effect of water concentration on photocatalytic coupling reactions. Reaction conditions: 10 mg photocatalysts in 2 mL 1 vol % water‐solvent solution with 8 mM reactant and 40 mM KOH under 410 nm irradiation (30 mW cm^−2^) and 1 bar N_2_ at RT. d) Estimated QEs of gCN−OH for different reactions (12 mW cm^−2^). e) Stability of the gCN−OH for the synthesis of bibenzyl.

Similarly, gCN−OH also catalyzes the pinacol‐type coupling of benzaldehyde into hydrobenzoin (≈80 % yield) and the dehalogenative C−C coupling of benzyl bromide into bibenzyl (≈90 % yield) in aprotic solvents (dioxane and DMF) with 1 vol % of water (Figures 5a and b). No product is observed under dark conditions in the presence of gCN−OH (Tables S4 and S5). In all cases, the pristine gCN exhibits negligible catalytic performance. We have also evaluated a set of semiconductor photocatalysts (i.e., TiO_2_, BiOBr_*x*_, and AgGaO_2_) for these reactions, but poor activity is observed in all cases (Figure S13). We have further explored the effect of water concentration on the photocatalytic performance of gCN−OH (Figure 5c). No product is observed for all three coupling reactions in the absence of water, confirming that hydrogen solely originates from water rather than from the catalyst or the solvent (Tables S2, S4 and S5). The yield of product for all three types of coupling reaction reaches an optimum at a water concentration of ≈5 mol % (equivalent to 1 vol %). Though the optimum operation window of water concentration varies for different reactions, high coordination of water by solvent molecules appears favorable in general, suggesting that isolated water molecules are more prone to be dissociated by weakening of intermolecular hydrogen bonds.[^4^, ^5^]

The quantum efficiencies (QEs) are estimated to be 1.4, 0.7 and 1.6 % for the photosynthesis of azoxybenzene, hydrobenzoin, and bibenzyl under 410 nm irradiation, respectively (Figure 5d, Figure S14 and Note S5). The turnover frequencies (TOF) are estimated to be 10.6, 7.1, and 7.1 h^−1^ for the N−N coupling, pinacol‐type C−C coupling, and dehalogenative C−C coupling, respectively. The gCN−OH photocatalyst displays a good stability for the synthesis of bibenzyl (Figure 5e), where no obvious decay of performance is observed for four consecutive cycles.

**Figure 6:**
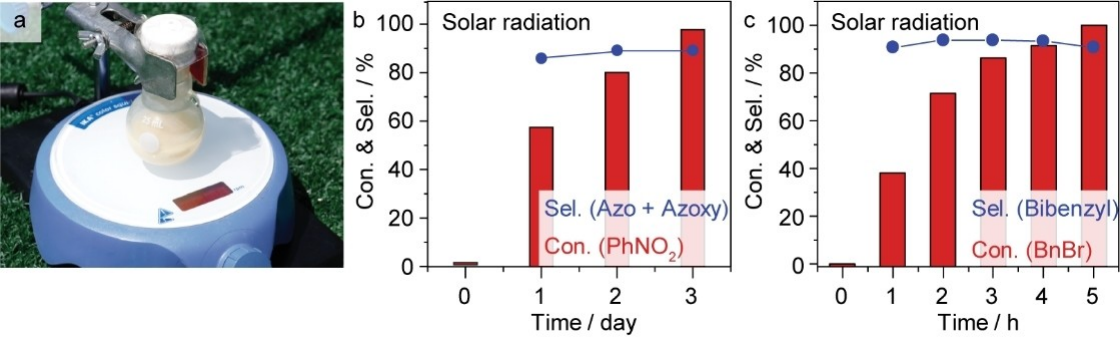
Photocatalytic performances under solar irradiation. a) Image of the reaction system. b), c) Hydrogenative homocoupling of nitrobenzene and benzyl bromide (8 mM reactant and 75 mg catalyst in 15 mL 1 vol % water‐dioxane solution).

Furthermore, the gCN−OH can dehydrogenate water under solar irradiation to drive the coupling reactions (Figure 6a, Figure S15). Nitrobenzene can be fully converted into the corresponding N−N compound within three days (Figure 6b). Meanwhile, the conversion of benzyl‐bromide into bibenzyl reaches completion within only 5 h (Figure 6c), possibly owing to a two‐electron process that is much easier than the N−N coupling of nitrobenzene (6 e^−^).

The gCN−OH photocatalyst displays satisfactory performance in expanding the substrates for N−N and C−C coupling employing water as the hydrogen donor under irradiation (Table 1). All nitrobenzene derivatives exhibit high conversions (**1** **a** to **1** **m**). The presence of either an electron donating group (i.e., −OCH_3_) or electron withdrawing group (i.e., −CF_3_) shows negligible impact on the selective synthesis of the corresponding azoxy aromatics (**1** **a** to **1** **h**), with only *p*‐methylnitrobenzene as an exception (**1** **c**). Likewise, the *m,m*′‐dichloro azoxybenzene can also be selectively synthesized from *m*‐chloro‐nitrobenzene (**1** **i**). The photocatalytic coupling of 3,5‐dimethyl‐ and 3,4‐dimethyl‐nitrobenzene (**1** **j** and **1** **k**) into corresponding azoxy‐aromatics show high conversion and selectivity, indicating that also more complicated azoxy compounds can be synthesized via this approach. Note also that pentachloroaniline (**1** **m**), rather than the azoxy compound, is the major product in photoconversion of quintozene, possibly due to the combination of strong electron withdrawing and steric effects.

**Table 1:**
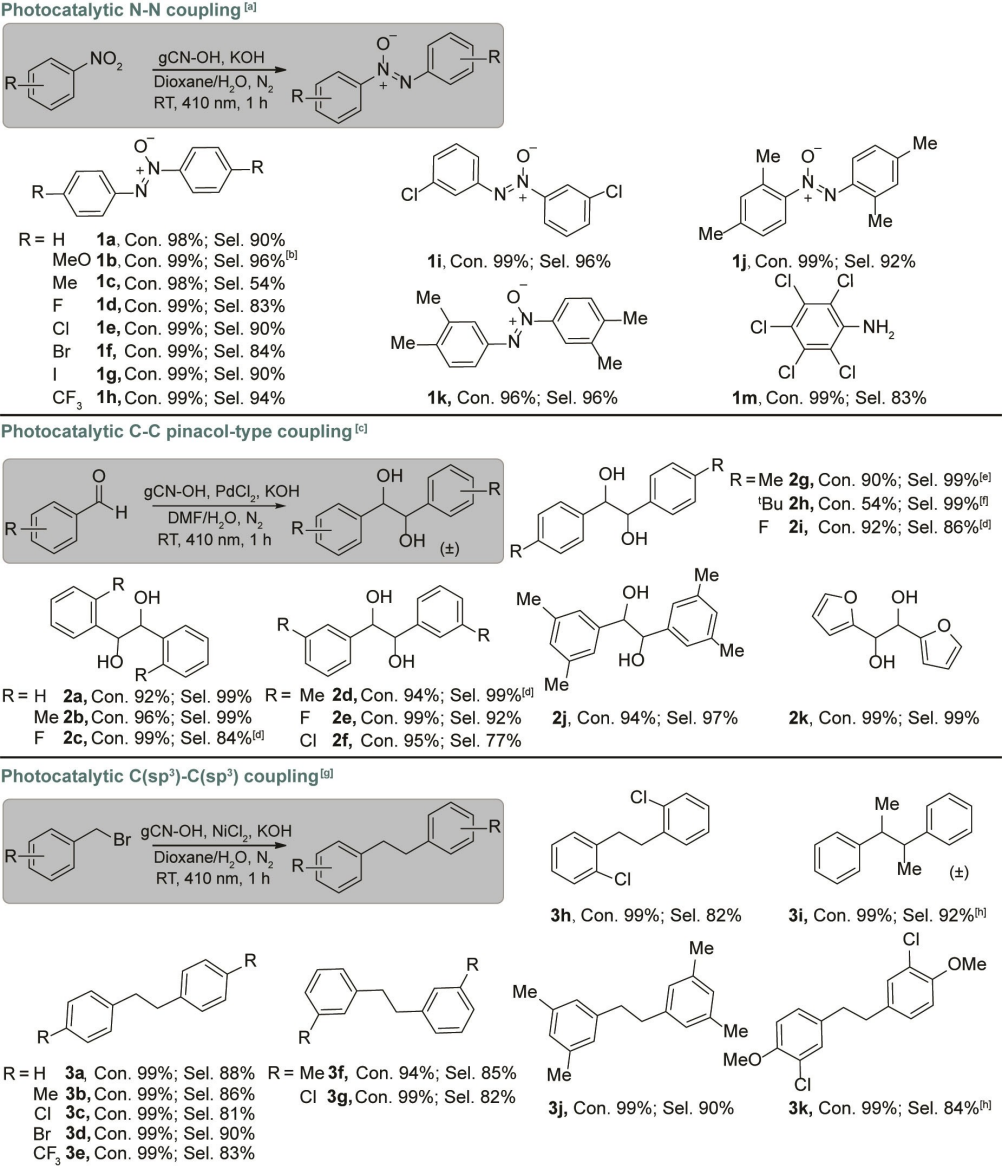
Substrate scope. Photocatalytic N−N coupling, pinacol‐type coupling and dehalogenative C−C coupling using gCN−OH with water as the hydrogen donor.

Reaction conditions: [a] 8 mM reactant, 10 mg gCN−OH and 4 mg KOH in 2 mL 1 vol % H_2_O–dioxane solution, 410 nm LED (30 mW cm^−2^), 1 bar N_2_ at RT for 1 h. [b] Same as [a], 450 nm LED (30 mW cm^−2^) for 3 h. [c] Same as [a], in 1 vol % H_2_O‐DMF solution with additional 0.17 mg PdCl_2_. [d] Same as [c], 0.25 h; [e] Same as [c], 0.017 mg PdCl_2_, 4 h. [f] Same as [c], 3 h. [g] Same as [a], with additional 0.22 mg NiCl_2_. [h] Same as [g], 2 h. Con.: conversion, Sel.: selectivity.

A series of aromatic aldehydes shows high conversion for the synthesis of the corresponding pinacols (**2** **a** to **2** **k**) with 4‐tert‐butylbenzaldehyde as an exception (**2** **h**). Benzaldehydes with functional groups on *ortho‐* (**2** **b** and **2** **c**), *meta‐* (**2** **d** to **2** **f**), and *para‐* (**2** **g** to **2** **i**) positions can be fully converted into the related pinacols with high selectivity. The photoconversion of di‐substituted benzaldehyde (**2** **j**) also presents a high selectivity towards the corresponding pinacol. Noticeably, furfural **(2** **k)**, which is a common product from lignocellulosic biomass,^[26]^ undergoes photocatalytic pinacol‐type C−C coupling to produce hydrofuroin with high conversion and selectivity (**2** **k**). This provides a tool for the synthesis of value‐added pinacols from biomass using water as the hydrogen donor under ambient conditions. For the synthesis of bibenzyl compounds, a series of *p‐* (**3** **a** to **3** **e**), *m‐* (**3** **f** and **3** **g**), and *o‐* (**3** **h**) substituted benzyl bromides can be fully converted with high selectivity. The formation of meso‐2,3‐diphenylbutane (**3** **i**) is also achieved with high selectivity via photocatalytic homocoupling of 1‐phenethyl bromide. We have further examined the photoconversion of two di‐substituted benzyl bromides (3,5‐dimethylbenzylbromide and 3‐chloro‐4‐methoxybenzyl bromide). The representative precursors are fully converted into their corresponding bibenzyls with high selectivity (**3** **j** and **3** **k)**, again confirming the wide applicability of the gCN−OH photocatalyst in using water for coupling reactions.

## Conclusion

In the present work we provide a strategy for the photoactivation of water under visible light and mild reaction conditions. By investigating the complete process of water dissociation into a thermally stable compound (H_2_O_2_), the most energy demanding step is found to be the H−O bond dissociation of the very first water molecule (5.1 eV). The surface hydroxylation of graphitic carbon nitride offers an effective route to overcome this energy barrier with a BDE_H‐O_ of 2.25 eV, which can be initiated under visible light (≈550 nm). We have further demonstrated that this converts water into a robust hydrogen donor for multiple hydrogenative coupling reactions under visible light irradiation, including N−N coupling of nitrobenzene, pinacol‐type coupling of aldehydes and dehalogenative C−C coupling of benzyl bromides. The gCN−OH photocatalyst presents durable catalytic performance over multiple cycles with high yield and efficiency for a broad range of reactions, thus providing a low‐cost and environmentally benign solution of using renewable energy for a cleaner process in dye, electronic, and pharmaceutical industries.

## Conflict of interest

The authors declare no conflict of interest.

1

## Supporting information

As a service to our authors and readers, this journal provides supporting information supplied by the authors. Such materials are peer reviewed and may be re‐organized for online delivery, but are not copy‐edited or typeset. Technical support issues arising from supporting information (other than missing files) should be addressed to the authors.

Supporting Information

## Data Availability

The data that support the findings of this study are available in the Supporting Information of this article.
