# Supplementary material for: Photocatalytic Abstraction of Hydrogen Atoms from Water Using Hydroxylated Graphitic Carbon Nitride for Hydrogenative Coupling Reactions
Source: Angew Chem Int Ed Engl. 2022 Apr 11;61(24):e202204256. doi: 10.1002/anie.202204256 (PMC9320934; doi:10.1002/anie.202204256)
Supplement: Supplementary file 1 — Supporting Information [file ANIE-61-0-s001.pdf]

Angewandte Chemie
Eine Zeitschrift der Gesellschaft Deutscher Chemiker

Supporting Information

Photocatalytic Abstraction of Hydrogen Atoms from Water Using Hydroxylated Graphitic Carbon Nitride for Hydrogenative Coupling Reactions

D. Zhang, P. Ren, W. Liu, Y. Li, S. Salli, F. Han, W. Qiao, Y. Liu, Y. Fan, Y. Cui, Y. Shen, E. Richards, X. Wen, M. H. Rummeli, Y. Li, F. Besenbacher, H. Niemantsverdriet, T. Lim, R. Su**

Table of Contents

1. Supplementary Notes……………………………………………………………………………………………………….. 3
2. Supplementary Tables………………………………………………………………………………………………………5
3. Supplementary Figures……………………………………………………………………………………………………... 9
4. Supplementary References………………………………………………………………………………………………… 42

Supplementary Notes

Note S1. Synthetic protocol of photocatalysts.
All reagents were used without further purification unless otherwise stated. **gCN** was synthesized by a typical pyrolysis method with urea as the precursor.[1] Urea powders were loaded in a 30 mL crucible with a lid and heated to 550 °C in a muffle oven with a heating rate of 5 °C·min^{-1} and kept in air for 3 h. By anti-solvent method, **gCN-OH** was synthesized by dispersing gCN (0.5 g) into supersaturated KOH (4 M, 1.1 mL) organic solvent (100 mL) under continuous stirring at RT for 12 h. The final product was collected, washed with deionized (DI) water and dried at 60 °C under vacuum for 12 h. For ss-NMR analysis, ^{15}N labeled gCN and gCN-OH were synthesized using identical protocol with ^{15}N labelled urea as the precursor.

Note S2. Calculation of hydroxyl content.
First, the normalized peak areas of O, C, NC_3 and K are estimated according to Eq. S1:

$$N_A = \frac{I_A}{S_i}$$

Eq. S1

where N_A is the normalized peak area, I_A is the peak area, S_i is the relative sensitivity factors. The relative sensitivity factors of C1s, N1s, O1s and K2p are 1, 1.8, 2.93 and 3.97, respectively, and obtained from the element library of CasaXPS.

Second, the relative atomic percent contents of O, C, NC_3 and K are calculated according to Eq.S2:

$$C_A = \frac{N_A}{\sum_i N_{A,i}} * 100\%$$

Eq. S2

where N_A is the normalized peak area, C_A is the relative atomic percent contents.

Third, Since a melem unit has a central nitrogen (NC_3), the estimated atomic ratio of OH: NC_3:K is the molar ratio of OH: NC_3:K, in other words,this is the hydroxyl (OH) content of gCN-OH.

Last, the hydroxyl mass fraction can be calculated according to Eq. S3

$$\omega_A = \frac{C_A \times M_A}{\sum_i C_{A,i} \times M_i}$$

Eq. S3

where ω_A is the mass fraction, C_A is the relative atomic percentage, M is the molar mass (OH:17, melem units: 202, K:39).

Note S3. Hydrogen atom transfer steps in the three photocatalytic reactions.

1. Photocatalytic N-N coupling of nitrobenzene.[2] Nitrobenzene is first reduced to nitrosobenzene (NBS) with two hydrogen atoms produced from photocatalytic partial water dehydrogenation. NBS is further reduced to N-phenylhydroxyamine (NPH) with two additional hydrogen atoms. NBS and NPH undergoes a condensation process to produce azoxybenzene.

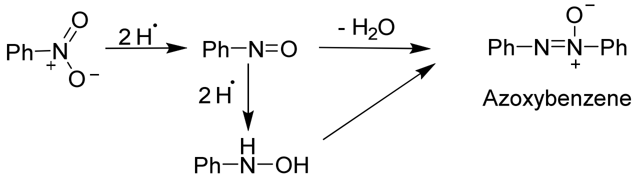

Eq. S4

2. Photocatalytic pinacol-type coupling of aldehydes.[3] Aromatic aldehyde is first reduced to a hydroxylated carbon centered radical with one hydrogen atom from water. The hydrobenzoin is produced via C-C homocoupling of the carbon radicals.

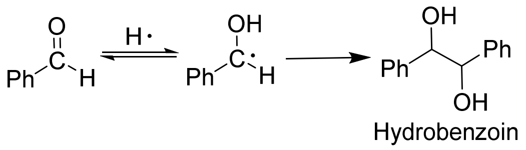

Eq. S5

3. Photocatalytic dehalogenative C-C coupling of benzyl bromides.[4] Benzyl bromide is dehalogenated into a benzyl radical and a bromine atom, which converts into HBr with a hydrogen atom that is abstracted from water. The benzyl radical undergoes a homocoupling in liquid phase to yield bibenzyl.

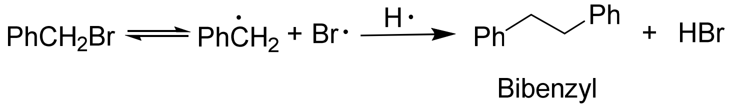

Eq. S6

Note S4. Estimation of H_2O consumption and H_2O_2 formation in photocatalytic nitrobenzene conversion.

The formation of nitroso benzene (NBS) and H_2O_2 from nitrobenzene consumes one part of H_2O (Eq. S7):

$$PhNO_2 + H_2O \longrightarrow PhNO + H_2O_2 \qquad \text{Eq. S7}$$

Converting NBS to N-phenylhydroxylamine (NPH) requires two parts of water with the formation of one part of H_2O_2 (Eq. S8):

$$PhNO + 2H_2O \longrightarrow PhNHOH + H_2O_2 \qquad \text{Eq. S8}$$

Therefore, three parts of water are needed to convert one nitrobenzene to NPH with two parts of H_2O_2 (Eq. S9):

$$PhNO_2 + 3H_2O \longrightarrow PhNHOH + 2H_2O_2 \qquad \text{Eq. S9}$$

NBS and NPH undergoes condensation coupling to produce azoxybenzene and water (Eq. S10):

$$PhNO + PhNHOH \longrightarrow Ph\text{-}\overset{O^-}{\overset{|}{N^+}}\text{=}N\text{-}Ph + H_2O \qquad \text{Eq. S10}$$

Therefore, the overall reaction from nitrobenzene to azoxybenzene can be simplified as the following (Eq. S11):

$$2PhNO_2 + 3H_2O \longrightarrow Ph\text{-}\overset{O^-}{\overset{|}{N^+}}\text{=}N\text{-}Ph + 3H_2O_2 \qquad \text{Eq. S11}$$

The theoretical amount of consumed H_2O and produced H_2O_2 in photocatalytic conversion of nitrobenzene can be calculated by the quantity of NBS, NPH, and azoxybenzene according to Eq. S12 and S13:

$$n_{\text{consumed water}} = 1 \times n_{NBS} + 3 \times n_{NPH} + 3 \times n_{azoxybenzene} \qquad \text{Eq. S12}$$

$$n_{\text{produced H2O2}} = 1 \times n_{NBS} + 2 \times n_{NPH} + 3 \times n_{azoxybenzene} \qquad \text{Eq. S13}$$

According to immediate GC analysis of the freshly irradiated photocatalyst-reactant suspension, 0.03 mmol of NBS, 0.12 mmol of NPH, and 0.11 mmol of azoxybenzene were produced, corresponding to a theoretical value of 0.72 mmol water consumed during photocatalytic nitrobenzene reduction. This matches well with the experimental value of consumed water by GC-MS (0.75 mmol).

According to GC analysis of the aliquots taken after UV-vis analysis (irradiation time of 2 h), 0.01 mmol of NBS, 0 mmol of NPH, and 0.05 mmol of azoxybenzene were produced, corresponding to a theoretical value of 0.16 mmol H_2O_2 produced during photocatalytic nitrobenzene reduction. The experimental value of produced H_2O_2 by UV-vis (0.12 mmol) is close to the theoretical value in consideration of H_2O_2 self-decomposition.

Note S5. Estimation of quantum efficiency (QE)
The QE of all three photocatalytic reactions were calculated according to Eq. S14

$$\mathrm{QE}=\frac{\sum_i \mathrm{Con.}*\mathrm{Sel.}*\mathrm{n_{reactant}}*\mathrm{n_{e,i}}}{\mathrm{N_{1h}}*\mathrm{t_R}}*100\% \qquad \text{Eq. S14}$$

where Con. is the conversion of a reaction at a given irradiation time (t_R), Sel. is the selectivity to target product, $n_{reactant}$ is the total number of reactant, $n_{e,i}$ is the number of electrons needed for converting one reactant to the corresponding product (3 for nitrobenzene, 1 for benzaldehyde, and 1 for benzyl bromide). The number of incident photons per hour (N_{1h}) is estimated according to Eq. S15:

$$N_{1\mathrm{h}} = \frac{E * \lambda * t}{h * c} = \frac{W_{lamp} * S_R * \lambda * t}{6.626 * 10^{-34} * 3 * 10^{8}} \qquad \text{Eq. S15}$$

where W_{lamp} is the light intensity of the LED lamp (0.012 $W \cdot cm^{-2}$), S_R is the effective irradiation area (12.56 cm^2), λ is the wavelength of the light (410 nm), t is irradiation time (3600 s), h is the Planck constant ($6.626 * 10^{-34}$ J·s) and c is the speed of the light ($3.0 * 10^{8}$ $m \cdot s^{-1}$). The estimated N_{1h} is $1.1*10^{21}$.

Supplementary Tables

Table S1. Calculated vibrational modes for 4-melem model with and without OH.

Models		Frequency / cm^{-1}	Vibrational mode description
4-melem model without OH	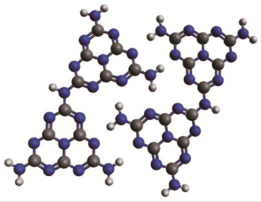	998	NH_2-NH_2 backbone
		1023	NH_2-NH_2 backbone
		1051	CN-CN backbone
4-melem model with OH on C next to NH_2	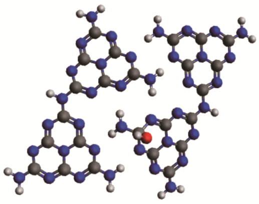	942	C-N-C-N-C backbone mode
		986	H_2N-C(-N)-N backbone mode
		1016	O-CN_3 backbone mode
		1031	H_2N-C-O backbone mode
		1041	NCNCN-CN backbone mode
		1049	C-N-C-N-C-N-C backbone mode
		1054	C-N(-C)-C-N backbone mode
4-melem model with OH on C between 2 NH_2	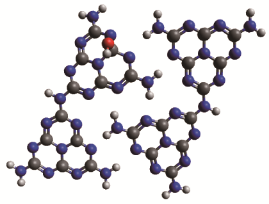	914	H-O-C-N-C backbone mode
		1002	C-N-C(-O)-N backbone mode
		1018	H-N(-H)-C-N backbone mode
		1040	C-N-C-N-C-N (triazine ring) backbone mode
		1050	CN-C(NH)C-CN backbone mode

Grey: C, blue: N, white: H, red: O.

Table S2. Control experiments for photocatalytic N-N coupling.[a]

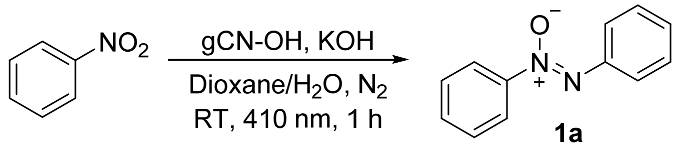

1a

Entry	Cat.	Conditions	**1a** (%)[b]
1	gCN-OH	Dioxane/H_2O (100:1 v/v), KOH, N_2, 410 nm	87
2	**NA**	Dioxane/H_2O (100:1 v/v), KOH, N_2, 410 nm	14
3	gCN-OH	**Dry** dioxane,[c] KOH, N_2, 410 nm	0
4	gCN-OH	Dioxane/H_2O (100:1 v/v), **non-base**, N_2, 410 nm	75
5	gCN-OH	Dioxane/H_2O (100:1 v/v), KOH, **Air**, 410 nm	58
6	gCN-OH	Dioxane/H_2O (100:1 v/v), KOH, N_2, **Dark**	0
7	gCN-OH	**DMF**/H_2O (100:1 v/v), KOH, N_2, 410 nm	35
8	gCN-OH	**CH_3CN**/H_2O (100:1 v/v), KOH, N_2, 410 nm	12
9	gCN	Dioxane/H_2O (100:1 v/v), **non-base**, N_2, 410 nm	3

[a] Reaction conditions: nitrobenzene (16 μmol), 10 mg photocatalyst in Dioxane (2 mL) at room temperature for 1 h under N_2. [b] GC yield. [c] Dry 1,4-dioxane, stored over activated molecular sieve 4Å for 3 days.

We have prepared the gCN-OH with a series of KOH concentrations and evaluated their photocatalytic performance (Table S3). The catalytic activity of the gCN-OH gradually improved upon increasing the KOH concentration and reached an optimum at 4 M, suggesting that a certain concentration of KOH is required to effectively hydroxylate the surface of gCN.

Table S3. Effect of KOH concentration during synthesis of gCN-OH and the catalytic performance of the corresponding gCN-OH.

Entry	Cat.	c(KOH) / M	Con. (%)	Sel. (%)
1	gCN-OH	0.01	11	90
2	gCN-OH	0.1	20	92
3	gCN-OH	0.5	43	89
4	gCN-OH	1	78	90
5	gCN-OH	4	98	88
6	gCN-OH	10	99	87

Reaction conditions: nitrobenzene (16 μmol), KOH, 10 mg photocatalyst in Dioxane (2 mL) at room temperature for 1 h under N_2.

Table S4. Control experiments for photocatalytic pinacol-type coupling.[a]

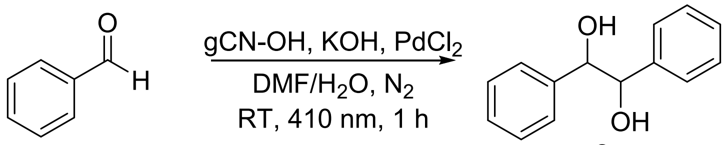

2a

Entry	Cat.	Conditions	**2a** (%)[b]
1	$PdCl_2$, gCN-OH	DMF/H_2O (100:1 v/v), KOH, N_2, 410 nm	91
2	**Non-$PdCl_2$**, gCN-OH	DMF/H_2O (100:1 v/v), KOH, N_2, 410 nm	34
3	$PdCl_2$, **non-photocat**	DMF/H_2O (100:1 v/v), KOH, N_2, 410 nm	0
4	$PdCl_2$, gCN-OH	**Dry** DMF[c], KOH, N_2, 410 nm	0
5	$PdCl_2$, gCN-OH	DMF/H_2O (100:1 v/v), **non-base**, N_2, 410 nm	0
6	$PdCl_2$, gCN-OH	DMF/H_2O (100:1 v/v), KOH, **Air**, 410 nm	13
7	$PdCl_2$, gCN-OH	DMF/H_2O (100:1 v/v), KOH, N_2, **Dark**	0
8	**$NiCl_2$**, gCN-OH	DMF/H_2O (100:1 v/v), KOH, N_2, 410 nm	11
9	**H_2PtCl_2**, gCN-OH	DMF/H_2O (100:1 v/v), KOH, N_2, 410 nm	9
10	**$HAuCl_4$**, gCN-OH	DMF/H_2O (100:1 v/v), KOH, N_2, 410 nm	11
11	$PdCl_2$, gCN-OH	**CH_3CN**/H_2O (100:1 v/v), KOH, N_2, 410 nm	0
12	$PdCl_2$, gCN-OH	**Dioxane**/H_2O (100:1 v/v), KOH, N_2, 410 nm	0

[a] Reaction conditions: benzaldehyde (16 μmol), 10 mg photocatalyst, $PdCl_2$ in DMF (2 mL) at room temperature for 1 h under N_2. [b] GC yield. [c] Dry DMF stored over activated molecular sieve 4Å for 3 days.

Table S5. Control experiments for photocatalytic dehalogenative C-C coupling.[a]

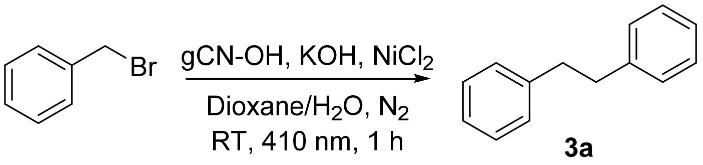

Entry	Cat.	Conditions	**3a** (%)[b]
1	$NiCl_2$, gCN-OH	CH_3CN/H_2O (100:1 v/v), KOH, N_2, 410 nm	79
2	**Non-$NiCl_2$**, gCN-OH	CH_3CN/H_2O (100:1 v/v), KOH, N_2, 410 nm	33
3	$NiCl_2$, **non-photocat.**	CH_3CN/H_2O (100:1 v/v), KOH, N_2, 410 nm	0
4	$NiCl_2$, gCN-OH	**Dry** CH_3CN,[c] KOH, N_2, 410 nm	0
5	$NiCl_2$, gCN-OH	CH_3CN/H_2O (100:1 v/v), **non-base**, N_2, 410 nm	27
6	$NiCl_2$, gCN-OH	CH_3CN/H_2O (100:1 v/v), KOH, **Air**, 410 nm	0
7	$NiCl_2$, gCN-OH	CH_3CN/H_2O (100:1 v/v), KOH, N_2, **Dark**	0
8	**$PdCl_2$**, gCN-OH	CH_3CN/H_2O (100:1 v/v), KOH, N_2, 410 nm	68
9	**H_2PtCl_2**, gCN-OH	CH_3CN/H_2O (100:1 v/v), KOH, N_2, 410 nm	38
10	**$HAuCl_4$**, gCN-OH	CH_3CN/H_2O (100:1 v/v), KOH, N_2, 410 nm	34
11	**$RuCl_3$**, gCN-OH	CH_3CN/H_2O (100:1 v/v), KOH, N_2, 410 nm	2
12	$NiCl_2$, gCN-OH	**DMF**/H_2O (100:1 v/v), KOH, N_2, 410 nm	47
13	$NiCl_2$, gCN-OH	**Dioxane**/H_2O (100:1 v/v), KOH, N_2, 410 nm	88

[a] Reaction conditions: benzyl bromide (16 μmol), 10 mg photocatalyst, $NiCl_2$ in Dioxane (2 mL) at room temperature for 1 h under N_2. [b] GC yield. [c] Dry acetonitrile stored over activated molecular sieve 4Å for 3 days.

Supplementary Figures

The binding energy of an OH^- ion onto crystalline gCN was estimated by subtracting the ΔH(gCN-OH model + OH^- ion far away) from the ΔH(gCN-OH hydroxyl ion attached), again using the same model system for the gCN crystalline structure stated in Fig. S1a and 1b. In addition, the binding energy of an overall neutrally bound hydroxyl radical on gCN was estimated in a similar way, using a neutral hydroxyl radical in place of OH^-, and was found to be slightly lower at 2.8 eV.

The structures for NMR calculations were adapted from the optimization calculations by MOPAC and the NMR chemical shift calculations were carried out with the gauge-including atomic orbitals (GIAO)[5] methodology implemented in the Gaussian 09 package[6] with the B3LYP exchange-correlation functional and 6-311+G(2d,p) basis set for all atoms. The references for calculating chemical shift from shielding constant were tetramethylsilane (TMS) for H, and NH_3 for N. All ^1H and ^{15}N NMR calculations are shown in Fig. S1c and 1d. The experimental values are listed for comparison.

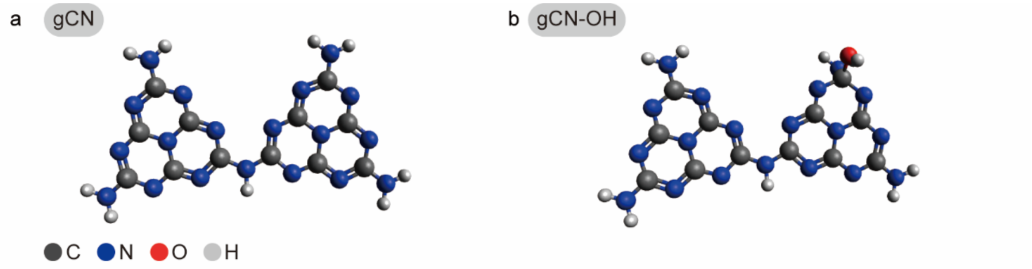

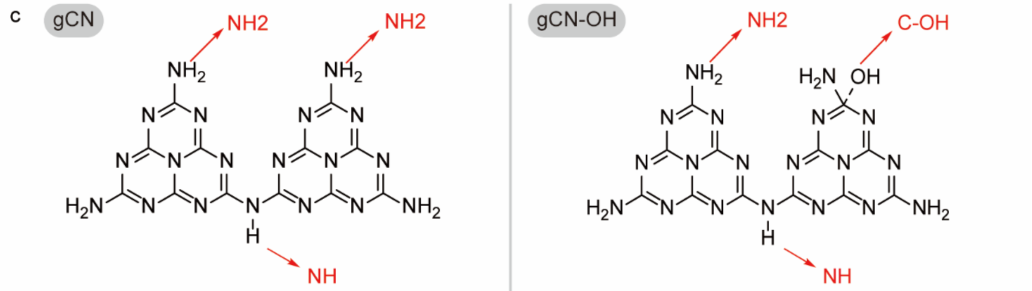

Sample	Site	δ(calc.) / ppm	δ(exp.) / ppm
gCN	H_{NH2}	5.24~5.40	3.89
	H_{NH}	9.16	9.13

Sample	Site	δ(calc.) / ppm	δ(exp.) / ppm
gCN-OH	H_{NH2}	5.07-5.32	3.36-4.45
	H_{NH}	9.30	9.16
	$H_{C\text{-}OH}$	7.38	6.91

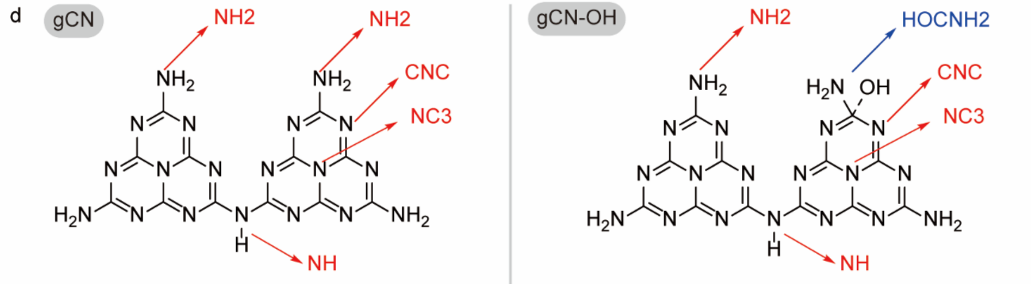

Sample	Site	δ(calc.) / ppm	δ(exp.) / ppm
gCN	N_{NH2}	99.9-100.5	114.6
	N_{NH}	164.2	134.7
	N_{NC3}	199.0-199.2	153.5
	N_{NCN}	228.5-246.7	191.8

Sample	Site	δ(calc.) / ppm	δ(exp.) / ppm
gCN-OH	N_{HOCNH2}	78.0	100.1
	N_{NH2}	82.9-95.2	116.4
	N_{NH}	169.2	134.7
	N_{NC3}	181.6-196.8	154.7
	N_{NCN}	214.1-253.9	191.0

Figure S1. ssNMR calculations**. a and b** Stabilized model structures of gCN and gCN-OH. **c and d** Comparison of calculated (calc.) and experimental (exp.) ^1H and ^{15}N ssNMR signal assignment for gCN and gCN-OH.

Chemical composition and oxidation state of elements on the surface region were characterized by an X-ray photoelectron spectrometer equipped with an Al Kα X-ray source (XPS, K-Alpha, Thermo Fisher Scientific, USA). Survey scans were measured from 1100 to -10 eV using a pass energy of 160 eV with a step size of 1 eV and a dwell time of 0.1 s, whereas the region-of-interest spectra of C1s, N1s, O1s and K2p were collected in the desired energy regions using a pass energy of 40 eV with a step size of 0.1 eV and a dwell time of 0.5 s. The C1s binding energy of adventitious carbon (284.6 eV) was used for binding energy calibration. Survey scans reveal that only C and N are observed on pristine gCN, whereas additional O and K exist in the gCN-OH (Fig. S2).

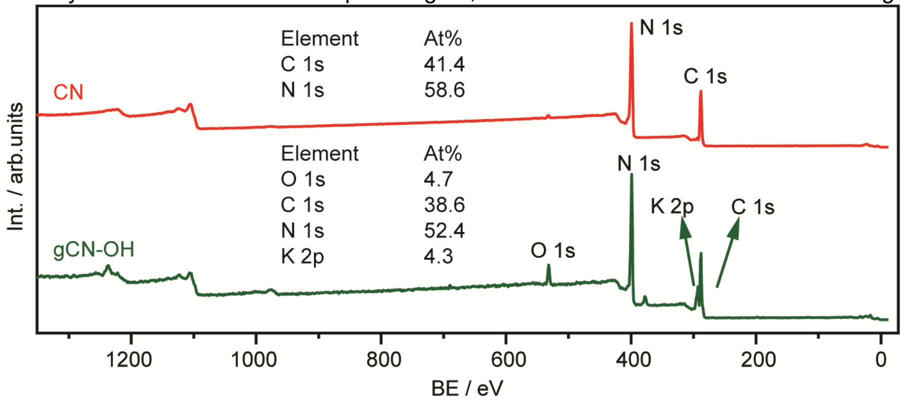

Figure S2. XPS survey spectra of the gCN and gCN-OH.

We have performed post-mortem analysis of the washed gCN-OH by XPS, as shown in Fig. S3. The oxygen peaks remain unchanged after 3 times of washing with DI water, confirming that the surface OH functional groups are attached to the catalyst with chemical bond rather than physical adsorption.

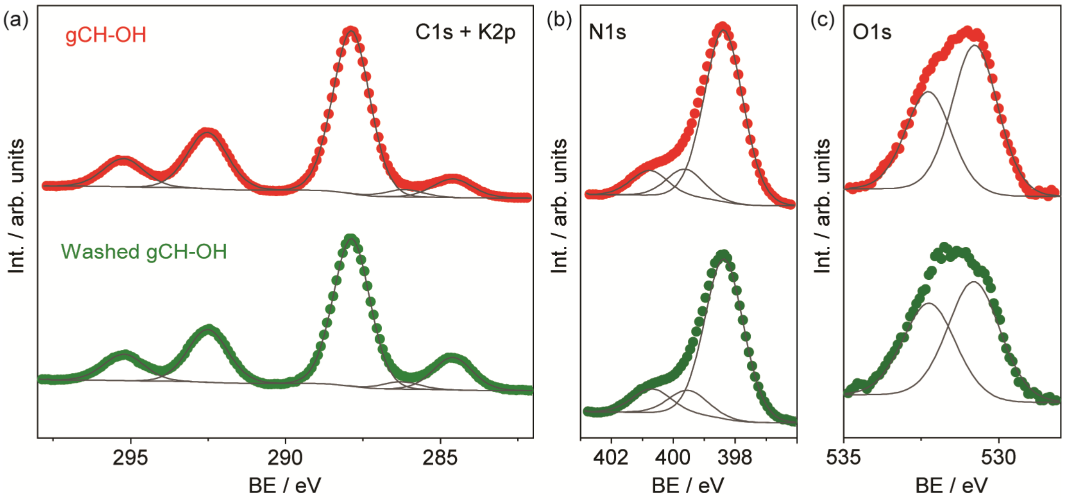

Figure S3. XPS spectra of the fresh gCN-OH and washed gCN-OH.

X-ray absorption spectroscopy (XAS) data were collected at the National Synchrotron Radiation Laboratory (NSRL, Beamlines MCD-A and MCD-B (Soochow Beamline for Energy Materials)). For pristine gCN, the distinct peak at ~288 eV is observed in the K-edge of C (Fig. S4a), which match well to the characteristic triazine structure.[7] An obvious shoulder is observed at ~287.2 eV for the gCN-OH that can be assigned to C–OH. In comparison, no obvious change of the K-edge of N is observed for gCN-OH (Fig. S4b), confirming that the surface hydroxyls are indeed bound to C.

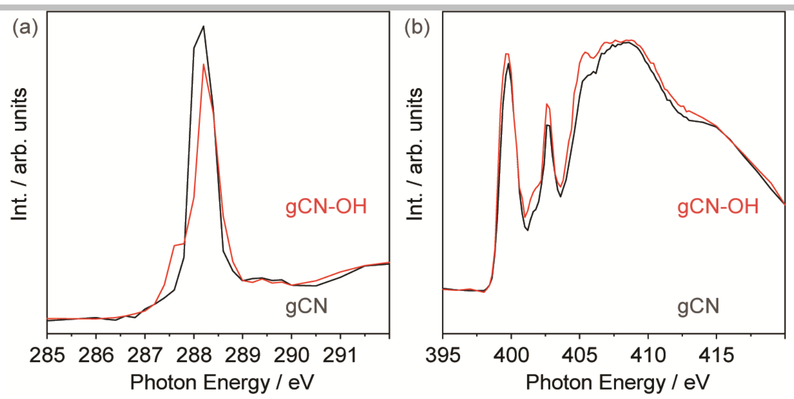

Figure S4. (a) and (b) C and N K edge XANES of gCN and gCN-OH.

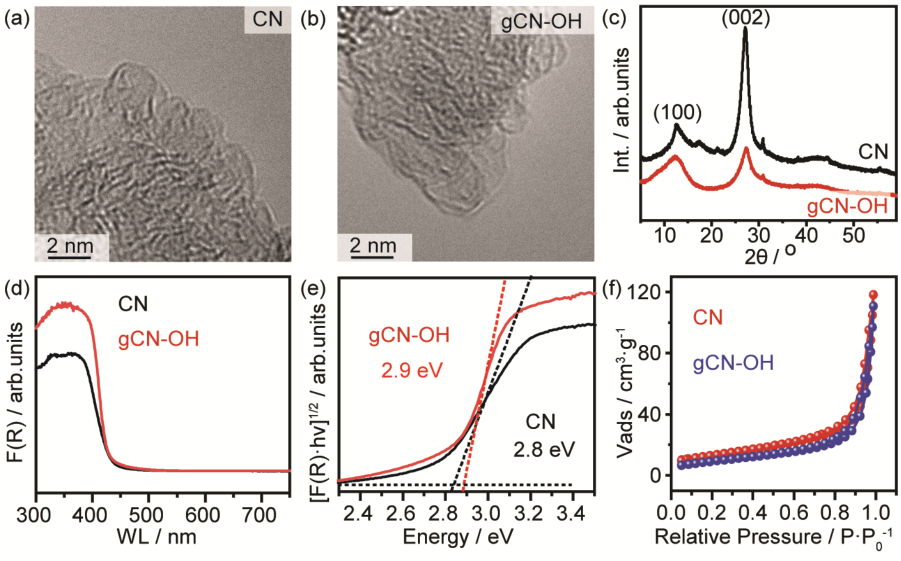

Figure S5. Characterization of gCN and gCN-OH**. a and b** TEM images. **c** PXRD patterns. **d and e** DRS and Tauc plots. **f** N_2 adsorption-desorption isotherms.

The morphology of gCN and gCN-OH was studied using a transmission electron microscope (TEM, Titan Themis Cubed G2 300). The powder samples were dispersed into ethanol and dropped on the Cu grid for analysis. As representatively shown in Figs. S5a and 5b, gCN and gCN-OH present a similar layered morphology.

X-ray diffraction (XRD) patterns were recorded on a Bruker D8 Advance diffractometer with a step size of 0.02° and a scan range of 5° to 60° using a Cu- Kα radiation source (40 kV, 40 mA). The XRD patterns of the gCN and gCN-OH show two characteristic peaks at 12.6° and 27.1°, corresponding to (100) and (002) facets of gCN, respectively (Fig. S5c).

Diffuse reflectance spectra (DRS) of the photocatalysts were measured using a Hitachi photospectrometer (UH4150). The DRS data were recorded in the range of 300-800 nm with spectroscopy grade $BaSO_4$ as the reference. The bandgap of the photocatalysts was derived using the Kubelka-Munk theory.[8] Supplementary Figures. 5d and 5e show DRS and Tauc plots of the gCN and gCN-OH.

The specific surface areas of gCN and gCN-OH were determined using a Micromeritics ASAP 2460 system, based on nitrogen adsorption/desorption isotherms measured at 77 K, as shown in Figure S5f. The specific surface area of gCN and gCN-OH are 46 and 34 $m^2 \cdot g^{-1}$, respectively. Both materials are non-porous.

Solid-state Nuclear Magnetic Resonance (ssNMR). All ^1H, ^{13}C and ^{15}N ssNMR spectra were recorded using an AVANCE III HD-600 MHz. The ^1H spectra were taken at a spinning speed of vR = 12.0 kHz, a 2.5 μs excitation pulse, a 10-s relaxation delay, and 32 scans. The CP/MAS ^{13}C NMR spectra were carried out using 1.4 ms contact time, 5 s relaxation delay, and 512 scans, respectively. The CP/MAS ^{13}C and ^{15}N NMR spectra were carried out using 3 ms contact time, 10 s relaxation delay, and 1024 scans, respectively. The ^{15}N-labeled gCN and gCN-OH sample were uniformly packed in a MAS rotor for ssNMR. The ^1H chemical shifts were referenced to adamantane at 1.91 ppm. The ^{13}C chemical shifts were referenced to the adamantane CH_2 peak at 38.48 ppm, and the ^{15}N chemical shifts were referenced to glycine at 32.0 ppm.

Photoluminescence spectra (PL) was recorded at RT using a fluorescence spectrometer (FLS1000) with a laser (λ = 320 nm). Attenuated total reflection infrared spectroscopy (ATR-IR) was performed using a Nicolet 6700 spectrometer.

The photochemical reactions were performed in a multichannel reactor (SUNCAT INSTRUMENTS, China) with blue light emitting diodes (LED, λ_{max} = 410 ± 10 nm). The setup consists of three parts: the LED source, a power supply, and a cooling system (Fig. S6). The liquid phase products were analyzed by gas chromatography (GC, Agilent 8860) and gas chromatography-mass spectrometry (GC-MS, Agilent 8860 network GC system coupled with 5977B Network Mass selective Detector). The GC system was equipped with a HP-5 column and an FID detector. The GC and GC-MS analyses were performed to determine the conversion and selectivity of the photocatalytic reactions. All reactions were carried out with solvents and reagents without further purification. For a typical reaction, 10 mg photocatalyst, 16 μmol reactant, 80 μmol KOH, and 20 μl DI water were added into 2 mL solvent at RT. The reactor was deaerated by N_2 purging prior to the photocatalytic reaction.

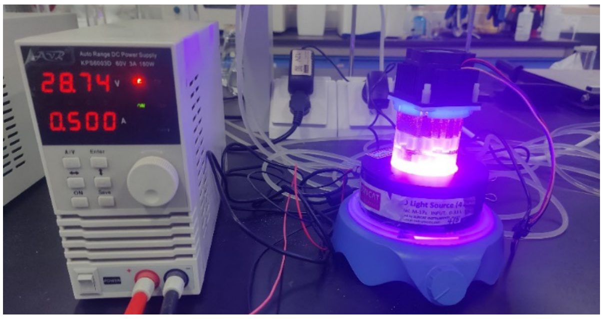

Figure S6. Photograph of the photocatalytic reaction setup.

Figure S7 shows the kinetic analysis of photocatalytic conversion of nitrobenzene, benzaldehyde, and benzyl bromide. All three reactions display pseudo-first-order kinetics with rate constants of 2.15 h^{-1}, 2.56 h^{-1}, and 2.81 h^{-1}, respectively.

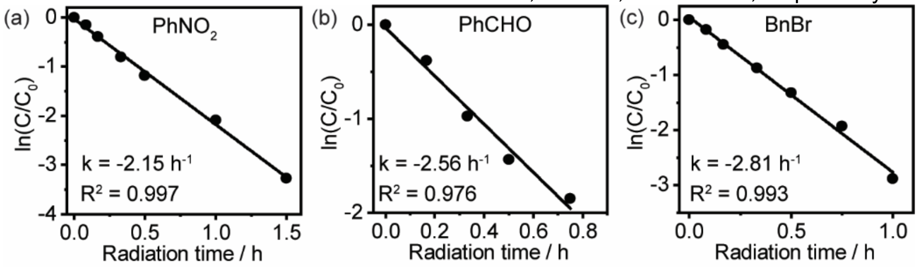

Figure S7. Reaction kinetic analysis**. a** $PhNO_2$, **b** PhCHO and **c** BnBr using gCN-OH under visible light irradiation. Reaction conditions: 10 mg photocatalysts in 2 ml water-solvent solution with 8 mM reactant and 40 mM KOH under 410 nm irradiation (30 mW·cm^{-2}) and 1 bar N_2 at RT.

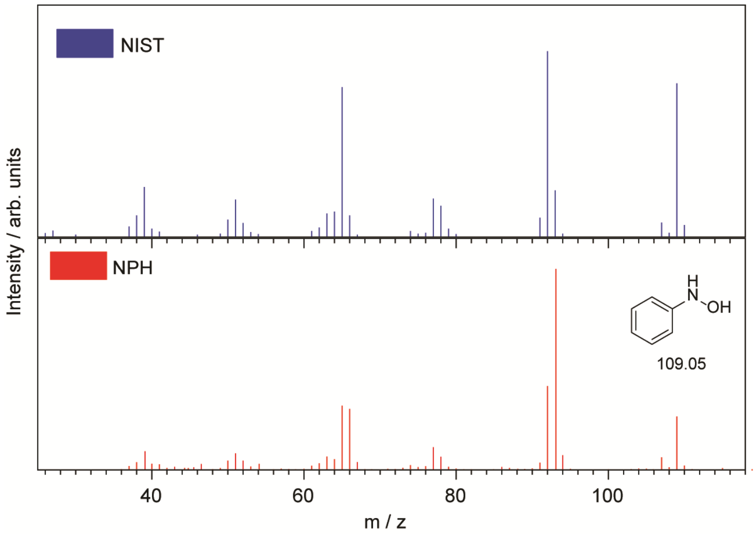

Figure S8. N-phenylhydroxyamine (NPH): the standard MS spectrum from the NIST database (above) and MS spectrum from GC-MS (below).

in-situ diffuse reflectance infrared Fourier transform spectroscopy (DRIFT) were measured *in-situ* using a Bruker VERTEX 70 FTIR spectrometer with a MCT detector.

The photogenerated H_2O_2 was quantified by a colorimetric titration method employing Cu (II) based chromogenic agent.[9] The chromogenic agent is prepared by mixing 2,9-dimethyl-1,10-phenanthroline (DMP) ethanol solution (1 g·L^{-1}) and aqueous $CuSO_4$ solution (0.01 M) in 1:1 volume ratio. For analysis, 50 μl of aliquots was added into 3 mL of the chromogenic agent under stirring for 10 min. The absorption spectra of the solution were then measured by a UV-vis spectrophotometer (UV 2600, Shimadzu), and the concentration of H_2O_2 was calculated using the absorbance at 454 nm by the Beer-Lambert law.

The absorption coefficient (α) of the reduced product, Cu (I)-DMP complex, is 1.79*10^7 M^{-1}·cm^{-1} according to the calibration curve (Fig. S9a). Note that one part of H2O2 can reduce two part of Cu(II)-DMP complex into Cu (I)-DMP complex. The UV-vis spectra for colorimetric titration of photogenerated H_2O_2 is shown in Fig. S9b, while the conversion of nitrobenzene determined by GC is shown in Fig. S9c. The evolution of H_2O_2 and nitrobenzene is shown in Fig. S9d. The Image of the aliquots taken from the photocatalytic reaction suspension mixed with Cu(II) based chromogenic agent is shown in Fig. S9e.

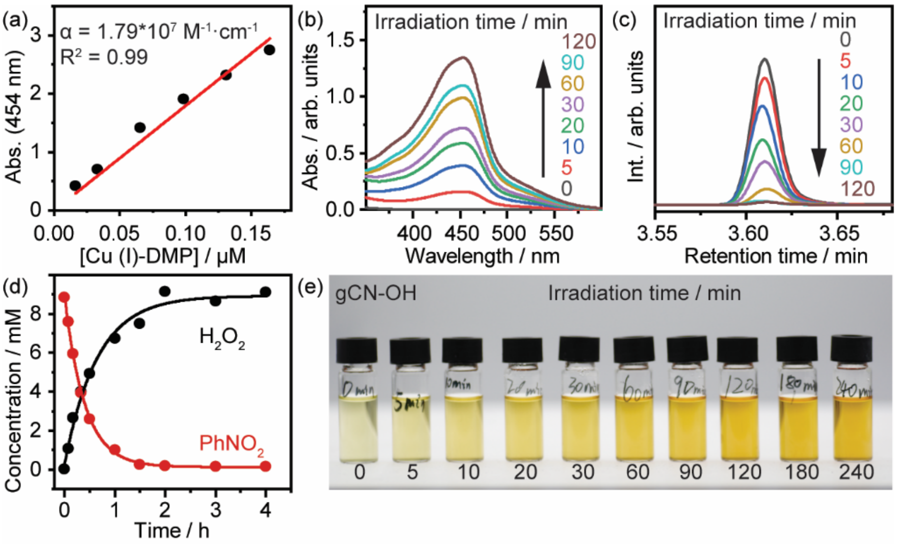

Figure S9. Colorimetric titration of H_2O_2 during photocatalytic N-N coupling**. a** Calibration curve for determining the absorption coefficient of Cu (I)-DMP complex. **b and c** UV-vis spectra of H_2O_2 formation and GC spectra of nitrobenzene conversion recorded during photocatalytic reaction at given irradiation time intervals. **d** Evolution of H_2O_2 and nitrobenzene derived from UV-vis and GC spectrometry. **e** Images of the aliquots taken from the photocatalytic reaction suspension mixed with Cu(II) based chromogenic agent.

Electron spin resonance (ESR). The room temperature X-band CW ESR measurements were performed by an Jeol JES-X320 spectrometer using 100 kHz field modulation frequency and 0.05 mT modulation width. ESR simulations were performed using the Easyspin toolbox. 20 mM of 5,5-dimethyl-1-pyrroline N-oxide (DMPO, spin trap) was added to the reaction suspension prior to irradiation, ensuring immediate capturing of all generated radicals for the formation of stable adducts. It should be noted that the estimated $t_{1/2}$ of the DMPO–•OOH adduct is ~ 45 s,[10] therefore irradiation times were limited to 30 s. Aliquots were removed from the reaction solution at the indicated times and filtered to remove all traces of photocatalyst prior to transfer to the ESR tube. Notably, this methodology does not enable accurate kinetic information to be extracted from the data (as the ESR signals are an accumulation of all radicals generated throughout the irradiation period), however this does increase the chance of trapping the full variety of radicals generated over the photocatalyst surfaces.

A series of control measurements were performed in the absence of photocatalyst to identify the presence of any radicals in the inert solvent system. The ESR spectra of the 1 vol% water-dioxane solution, both with and without KOH as a base, and under dark and irradiated conditions are shown in Fig. S10, employing DMPO as the spin trap. A negligible quantity of radical species was observed without photocatalyst in all cases.

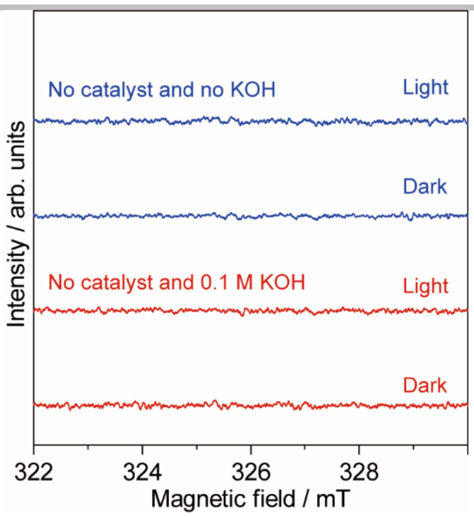

Figure S10. ESR spectra. 1 vol% water-dioxane solution in the presence of DMPO spin trap (blue lines), plus the addition of KOH (red lines) recorded at 298 K.

Semi-empirical molecular orbital geometry optimizations and transition state (TS) calculations were done using MOPAC2016, using the PM7 Hamiltonian.[11] The TS calculations for desorption of H_2O_2 were obtained using the SADDLE procedure outlined in the MOPAC2016 Manual. The bond dissociation energies estimated in this study were obtained by calculating the energy of undissociated water molecules and their resulting OH and H radicals after dissociating on a model gCN crystalline structure consisting of 2 strips of H-bonded 2-melem strips (Fig. S11), using the PM7 Hamiltonian in MOPAC2016. For the dissociation of a water molecule on the pristine gCN model, the BDE was estimated by subtracting the calculated ΔH of the clean model and a water molecule far away from the model surface, from the ΔH of an adsorbed OH on the model (effectively a model of gCN-OH) with a H radical far away from the surface. For the estimation of the BDE arising from a second dissociative attachment of OH onto gCN-OH, the BDE was estimated by correspondingly subtracting the ΔH(gCN-OH model + H_2O molecule far away) from the ΔH(gCN-OH with 2nd OH adsorbed + H radical far away). The resulting gCN model with 2 adsorbed adjacent OH radicals formed the start state for the TS calculation of the desorption of H_2O_2 from this model surface of gCN.

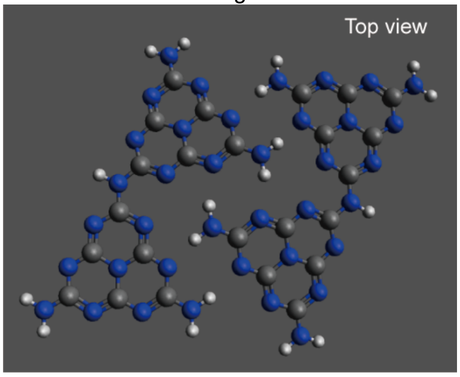

Figure S11. Calculated blank model.

All states used to estimate BDE are shown in Figure S12. There is little difference whether the OH is attached to the two different carbon sites.

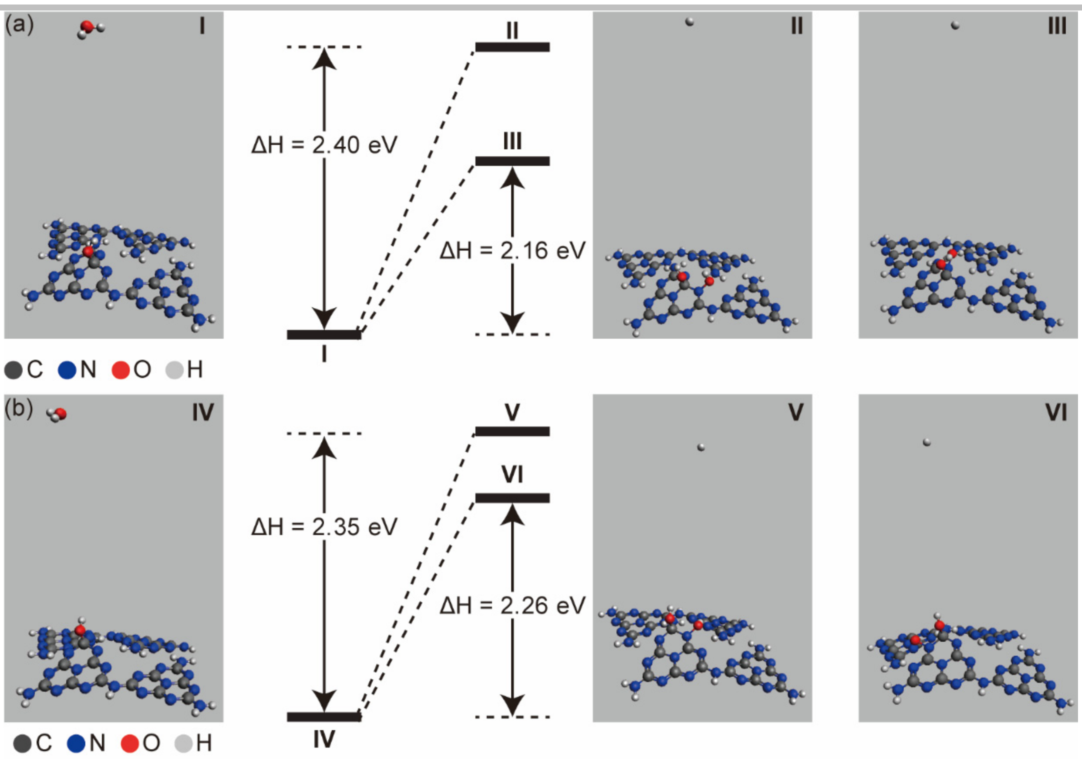

Figure S12. Calculated model states and H-O bond dissociation energy (BDE).

Figure S13 compares a series of typical photocatalysts for the N-N coupling, pinacol-type coupling, and dehalogenative C-C coupling. BiOBr, TiO_2, $AgGaO_2$, and NiFe-LDH show negligible activity for all reactions, whereas the BiVO4 shows poor performance for the synthesis of azoxybenzene and hydrobenzoin. In general, only gCN-OH shows satisfactory performance for all three reactions.

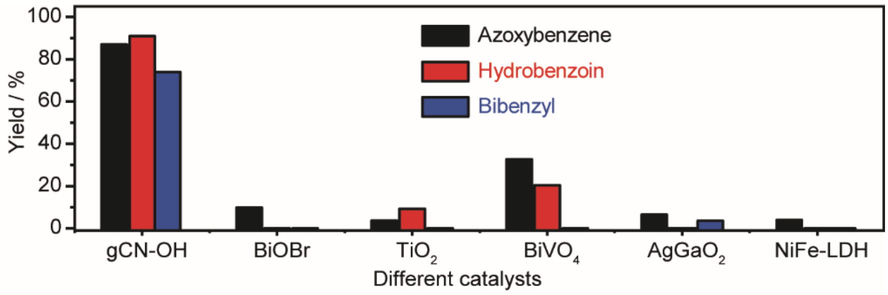

Figure S13. Survey of different photocatalysts for coupling reactions using water as hydrogen donor. Reaction conditions: 10 mg photocatalysts in 2 ml water-solvent solution with 8 mM reactant and 40 mM KOH under 410 nm irradiation (LED, 30 mW·cm^{-2}) and 1 bar N_2 at RT.

The QE of the three photocatalytic reaction was determined using a leak tight reactor that is connected to a stainless-steel vacuum/gas line, as reported previously (Fig. S14).[4] The reactor (A) is linked to the vacuum line (C) *via* a gas valve (D). The LED lamp (E) is placed on top of the quartz window (B).

An amount of 50 mg of photocatalyst and 8 mM of reactant were added into the 1 vol% H_2O-solvent solution. The suspension was dispersed by sonication. Before irradiation, the reactor was evacuated and filled with nitrogen three times. Then the suspension was irradiated by a 410 nm LED (12 mW·cm^{-2}).

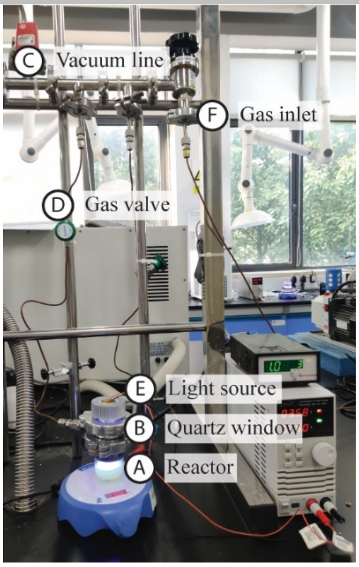

Figure S14. Setup for QE analysis.

The solar-driven photocatalytic coupling reaction was performed using a 25 mL round-bottom flask with leak-tight seals (Fig. S15). 75 mg of gCN-OH and 8 mM of reactant were dispersed in 15 mL of 1 vol% H_2O-solvent solution, which was purged by N_2 for 5 min prior to irradiation. The light intensity is recorded every hour for reference.

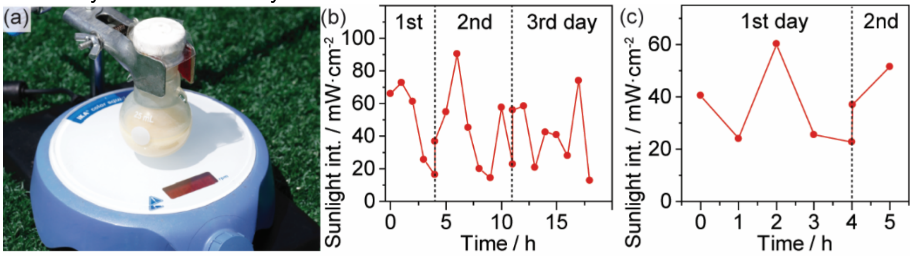

Figure S15. Setup and light intensity for solar driven photocatalytic reaction**.** **a** Photograph of photocatalytic nitrobenzene under solar radiation. The volume of the reaction suspension is 15 mL. **b** Sunlight intensity recorded during photocatalytic nitrobenzene conversion (Suzhou, 29/7/2020-31/7/2020, 27-34°C). **c** Sunlight intensity recorded during photocatalytic benzyl bromide conversion (Suzhou, 03/7/2021-03/7/2021, 25-30°C).

Detailed product analyses are shown in Supplementary Figures 16-64.

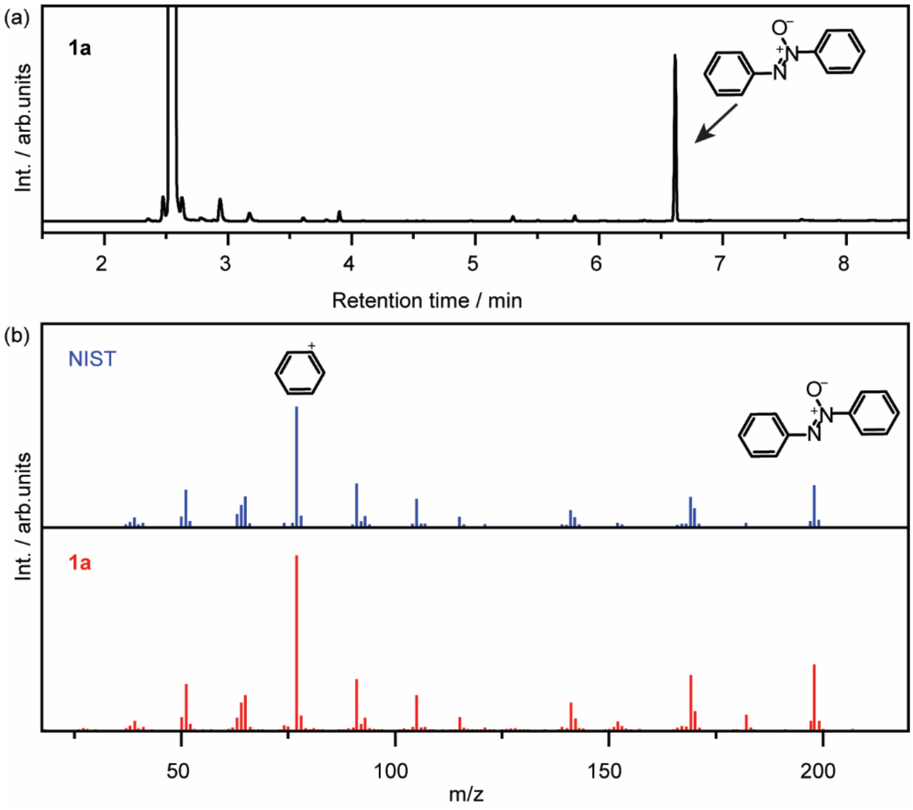

Figure S16. 1,2-diphenyldiazene 1-oxide **1a**: (a) GC spectrum. (b) MS spectrum from GC-MS (above) and the standard MS spectrum from the National Institute of Standards and Technology (NIST) database (below).

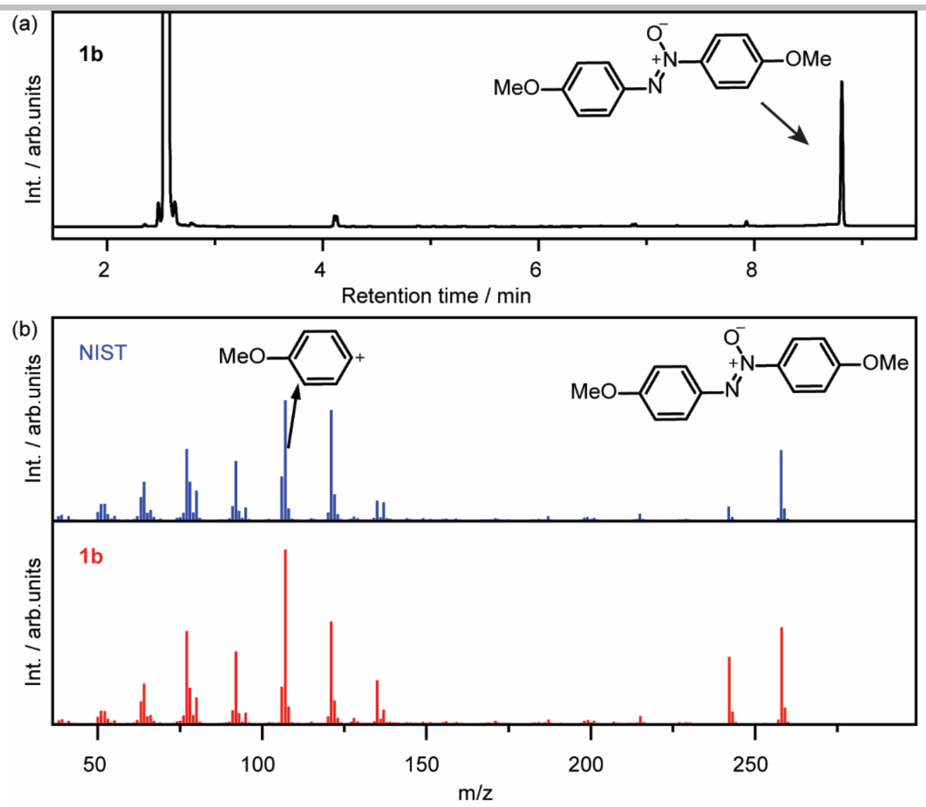

Figure S17. 1,2-bis(4-methoxyphenyl)diazene 1-oxide **1b**: (a) GC spectrum. (b) MS spectrum from GC-MS (above) and the standard MS spectrum from the NIST database (below).

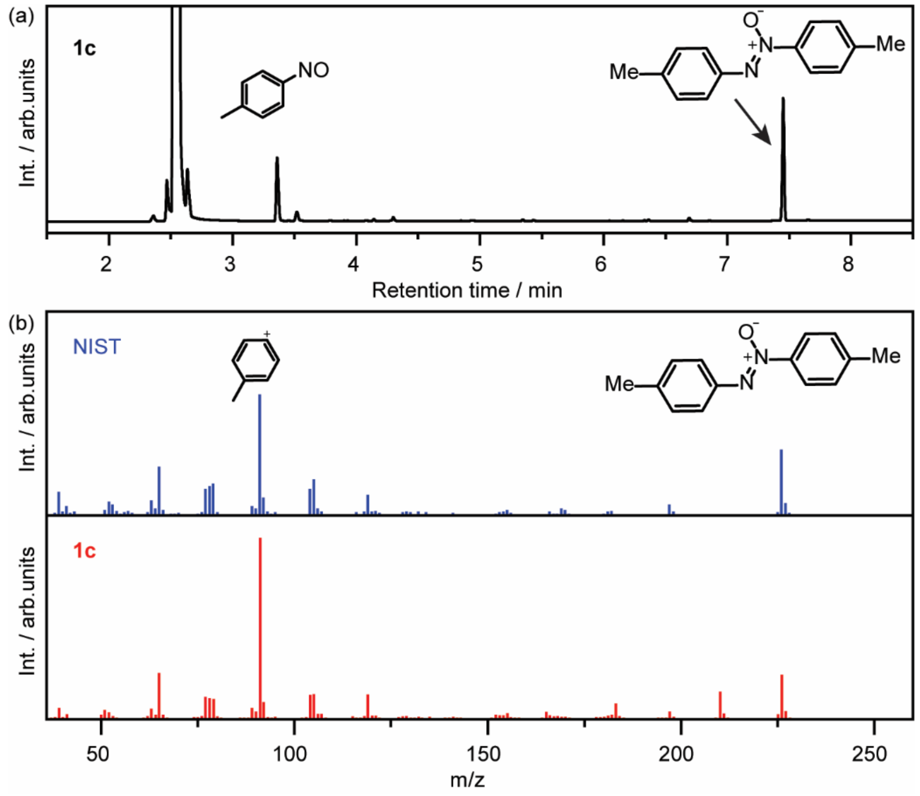

Figure S18. 1,2-di-*p*-tolyldiazene 1-oxide **1c**: (a) GC spectrum. (b) MS spectrum from GC-MS (above) and the standard MS spectrum from the NIST database (below).

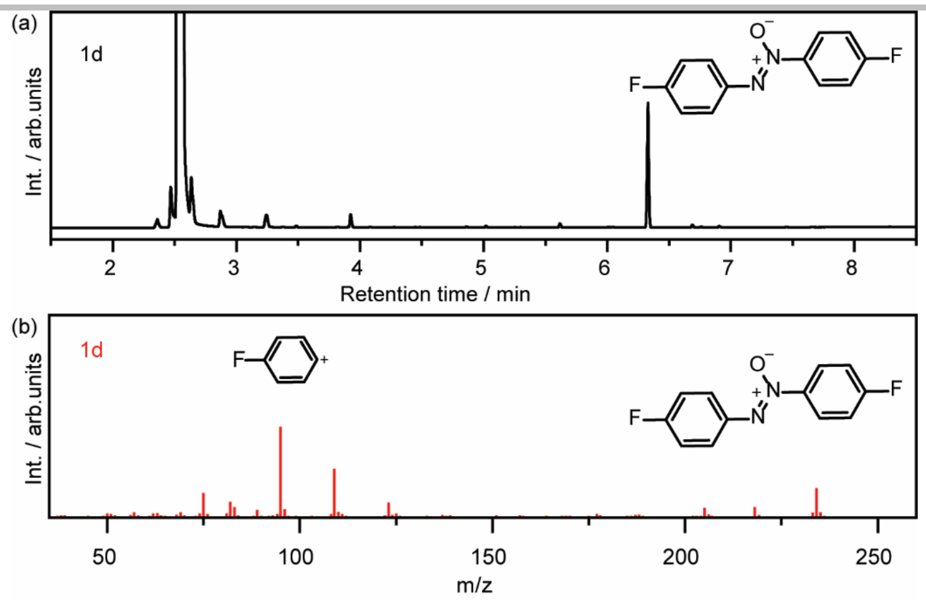

Figure S19. 1,2-bis(4-fluorophenyl)diazene 1-oxide **1d**: (a) GC spectrum. (b) MS spectrum from GC-MS.

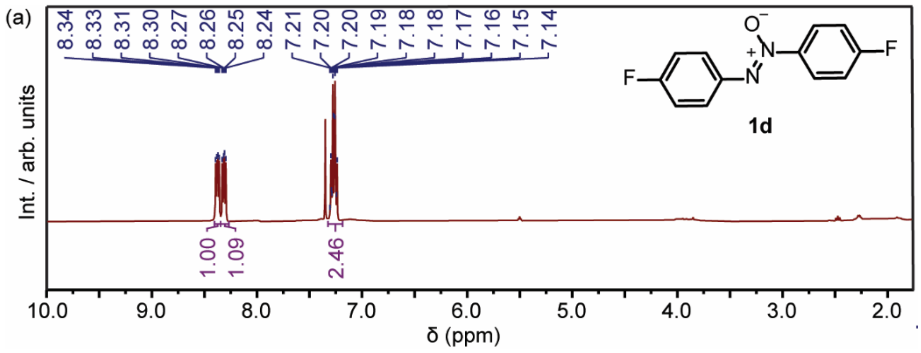

Figure S20. (a) ^{1}H-NMR spectra of **1d**. ^{1}H NMR (400 MHz, $CDCl_3$) δ 8.35 – 8.29 (m, 1H), 8.29 – 8.21 (m, 1H), 7.23 – 7.09 (m, 2H). The spectroscopic data match the previously reported data.[12]

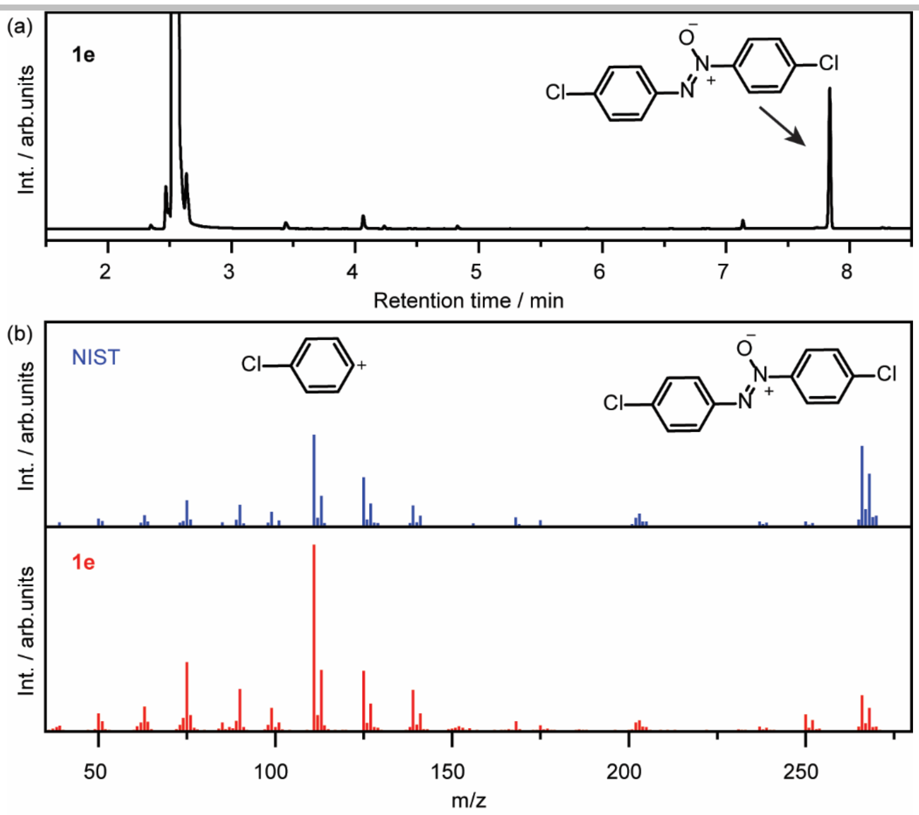

Figure S21. 1,2-bis(4-chlorophenyl)diazene 1-oxide **1e**: (a) GC spectrum. (b) MS spectrum from GC-MS (above) and the standard MS spectrum from the NIST database (below).

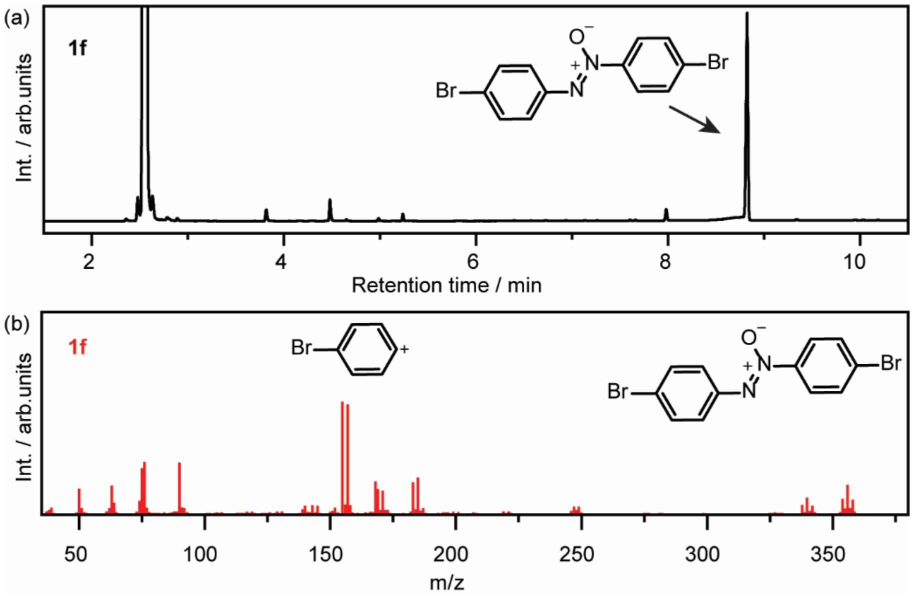

Figure S22. 1,2-bis(4-bromophenyl)diazene 1-oxide **1f**: (a) GC spectrum. (b) MS spectrum from GC-MS.

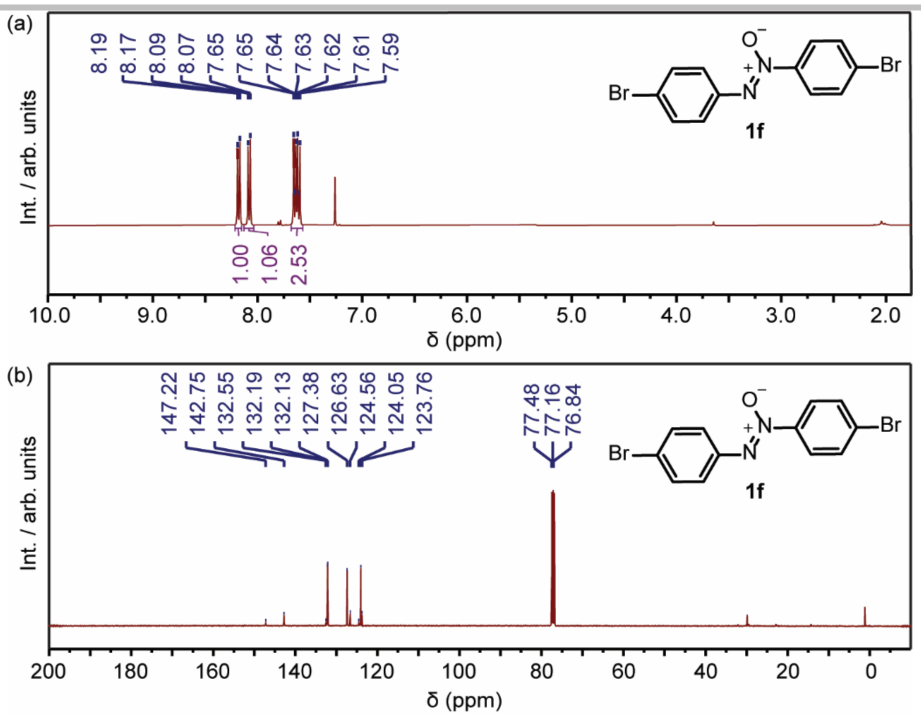

Figure S23. NMR spectra of **1f**. (a) ^{1}H NMR (400 MHz, $CDCl_3$) δ 8.18 (d, *J* = 9.0 Hz, 1H), 8.08 (d, *J* = 8.9 Hz, 1H), 7.68 – 7.57 (m, 3H). (b) ^{13}C NMR (101 MHz, $CDCl_3$) δ 147.2, 142.7, 132.5, 132.2, 132.1, 127.4, 126.6, 124.6, 124.1, 123.8.

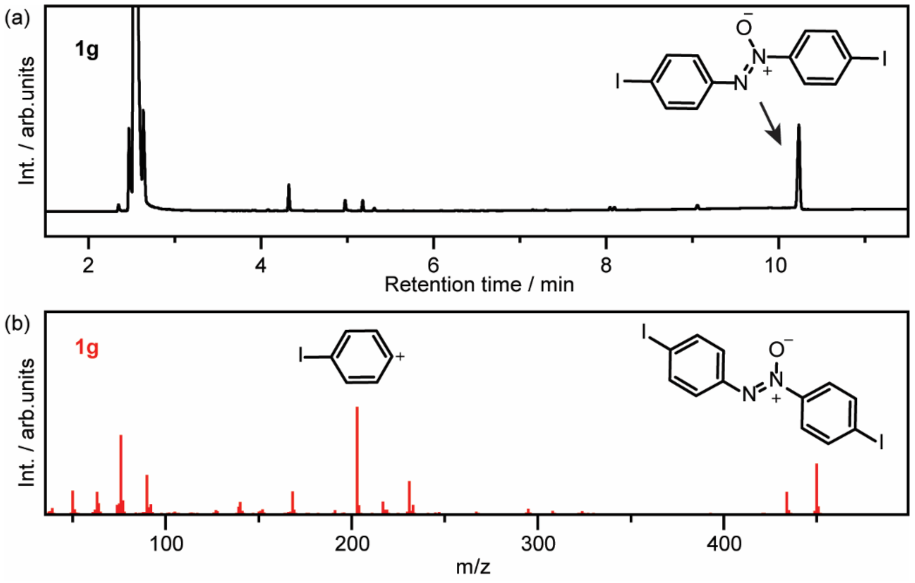

Figure S24. 1,2-bis(4-iodophenyl)diazene 1-oxide **1g**: (a) GC spectrum. (b) MS spectrum from GC-MS.

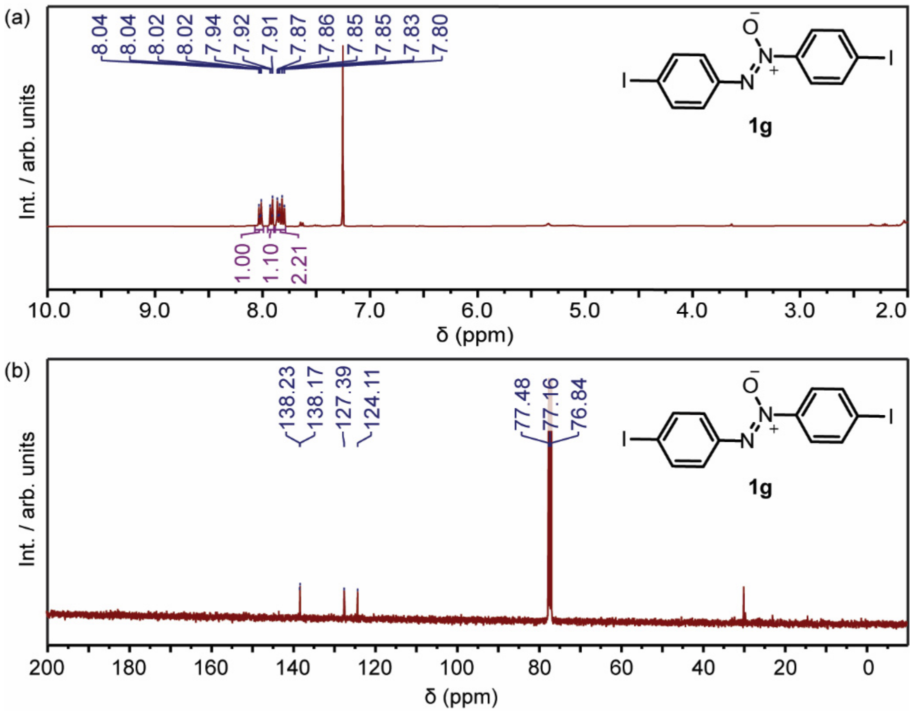

Figure S25. NMR spectra of **1g**. (a) ^{1}H NMR (400 MHz, $CDCl_3$) δ 8.08 – 8.00 (m, 1H), 7.92 (d, *J* = 8.8 Hz, 1H), 7.89 – 7.79 (m, 2H). (b) ^{13}C NMR (101 MHz, $CDCl_3$) δ 138.2, 138.2, 127.4, 124.1.

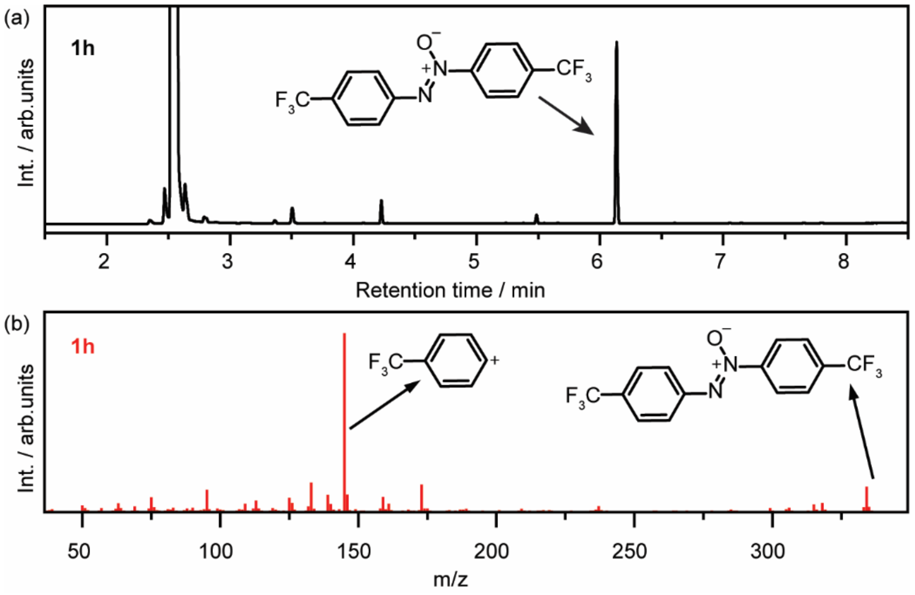

Figure S26. 1,2-bis(4-(trifluoromethyl)phenyl)diazene 1-oxide **1h**: (a) GC spectrum. (b) MS spectrum from GC-MS (above) and the standard MS spectrum from the NIST database (below).

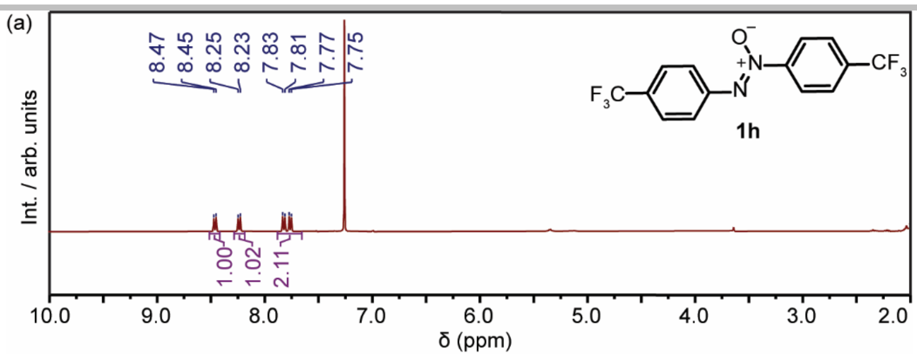

Figure S27. NMR spectra of **1g**. (a) ^{1}H NMR (400 MHz, $CDCl_3$) δ 8.46 (d, *J* = 8.5 Hz, 1H), 8.24 (d, *J* = 8.4 Hz, 1H), 7.79 (dd, *J* = 23.9, 8.5 Hz, 2H). The spectroscopic data match the previously reported data.[13]

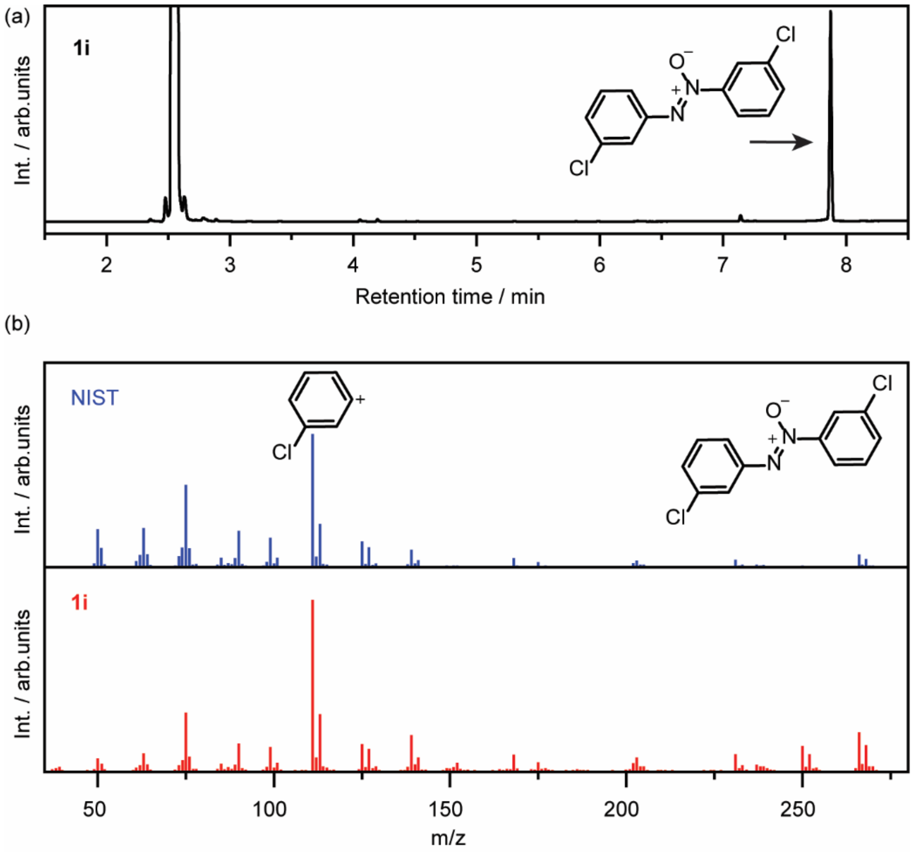

Figure S28. 1,2-bis(3-chlorophenyl)diazene 1-oxide **1i**:(a) GC spectrum. (b) MS spectrum from GC-MS (above) and the standard MS spectrum from the NIST database (below).

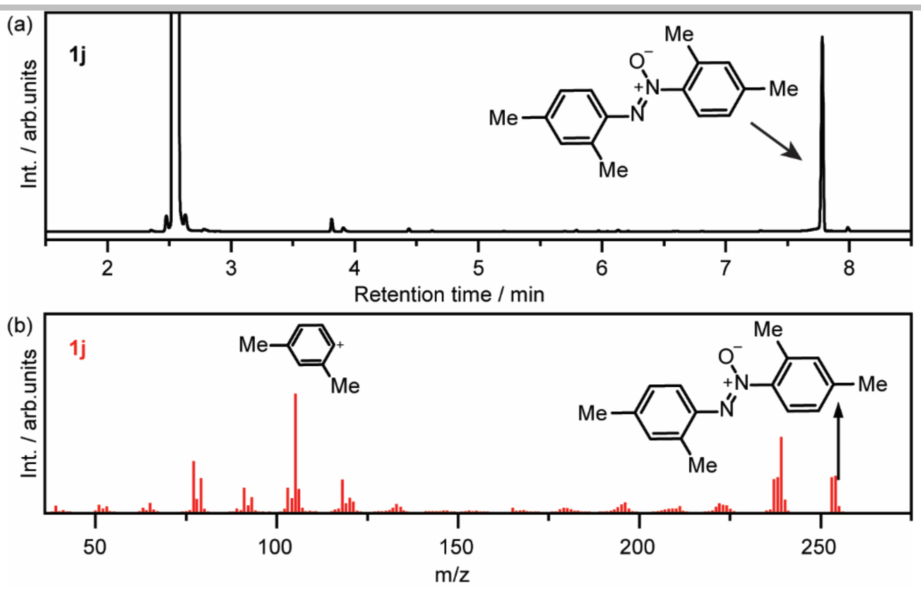

Figure S29. 1,2-bis(2,4-dimethylphenyl)diazene 1-oxide **1j**:(a) GC spectrum. (b) MS spectrum from GC-MS (above) and the standard MS spectrum from the NIST database (below).

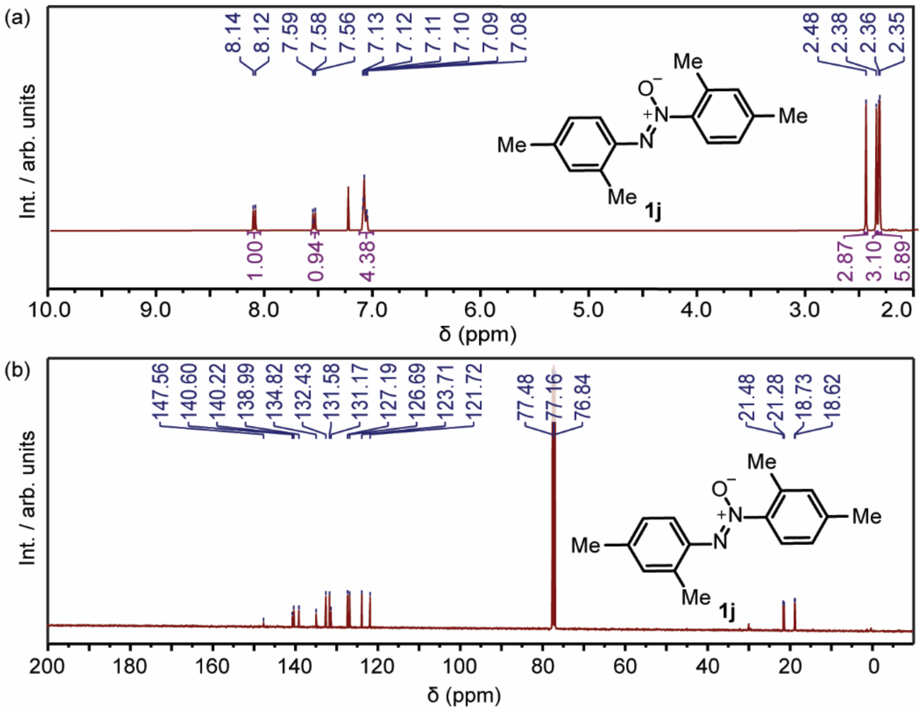

Figure S30. NMR spectra of **1j.** (a) ^{1}H NMR (400 MHz, $CDCl_3$) δ 8.13 (d, *J* = 8.2 Hz, 1H), 7.58 (d, *J* = 8.5 Hz, 1H), 7.16 – 7.03 (m, 4H), 2.48 (s, 3H), 2.38 (s, 3H), 2.36 (d, *J* = 3.1 Hz, 6H). (b) ^{13}C NMR (101 MHz, $CDCl_3$) δ 147.6, 140.6, 140.2, 139.0, 134.8, 132.4, 131.6, 131.2, 127.2, 126.7, 123.7, 121.7, 21.5, 21.3, 18.7, 18.6.

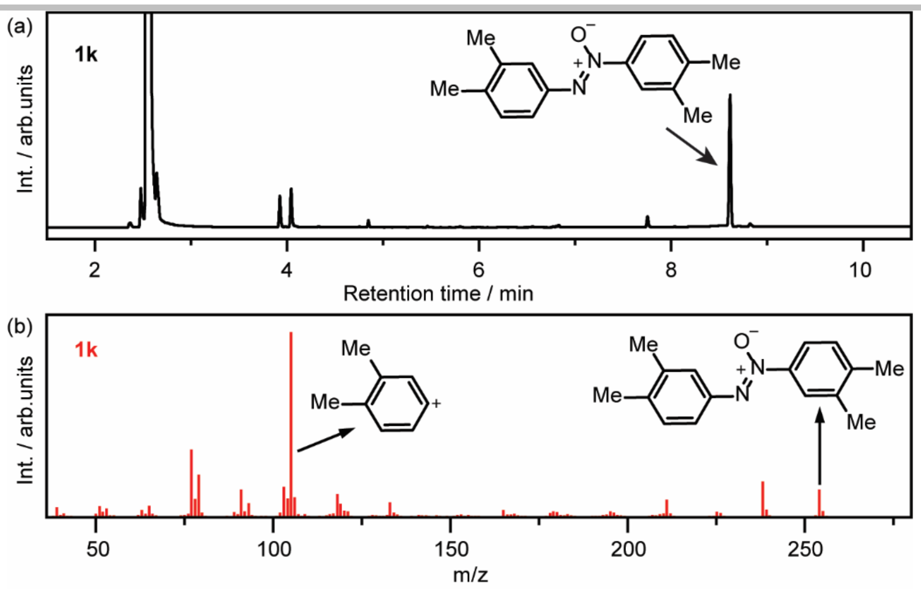

Figure S31. 1,2-bis(3,4-dimethylphenyl)diazene 1-oxide **1k**: (a) GC spectrum. (b) MS spectrum from GC-MS.

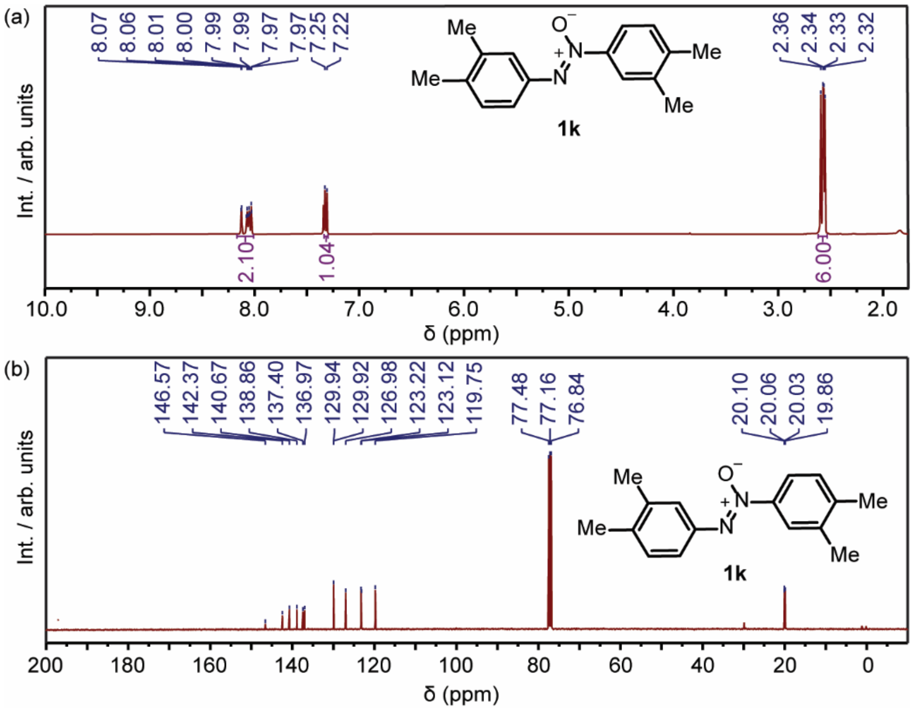

Figure S32. NMR spectra of **1k**. (a) ^{1}H NMR (400 MHz, $CDCl_3$) δ 8.11 – 7.95 (m, 2H), 7.23 (d, *J* = 8.2 Hz, 1H), 2.39 – 2.30 (m, 6H). (b) ^{13}C NMR (101 MHz, $CDCl_3$) δ 146.6, 142.4, 140.7, 138.9, 137.4, 137.0, 129.9, 129.9, 127.0, 123.2, 123.1, 119.8, 20.1, 20.1, 20.0, 19.9.

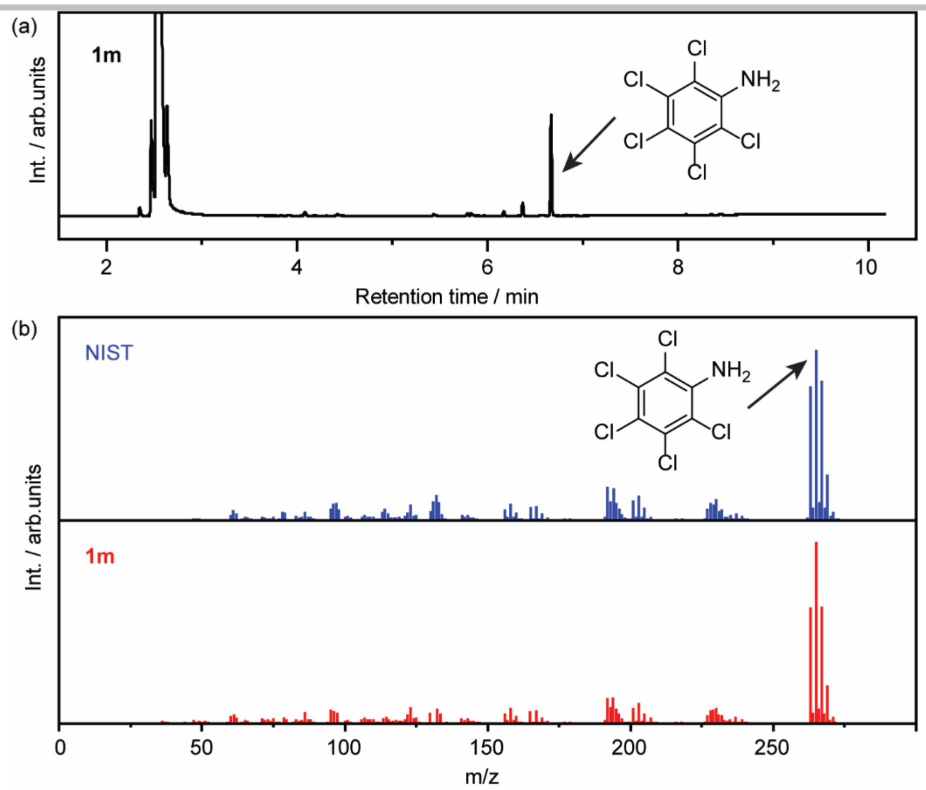

Figure S33. 2,3,4,5,6-pentachloroaniline **1m**: (a) GC spectrum. (b) MS spectrum from GC-MS (above) and the standard MS spectrum from the NIST database (below).

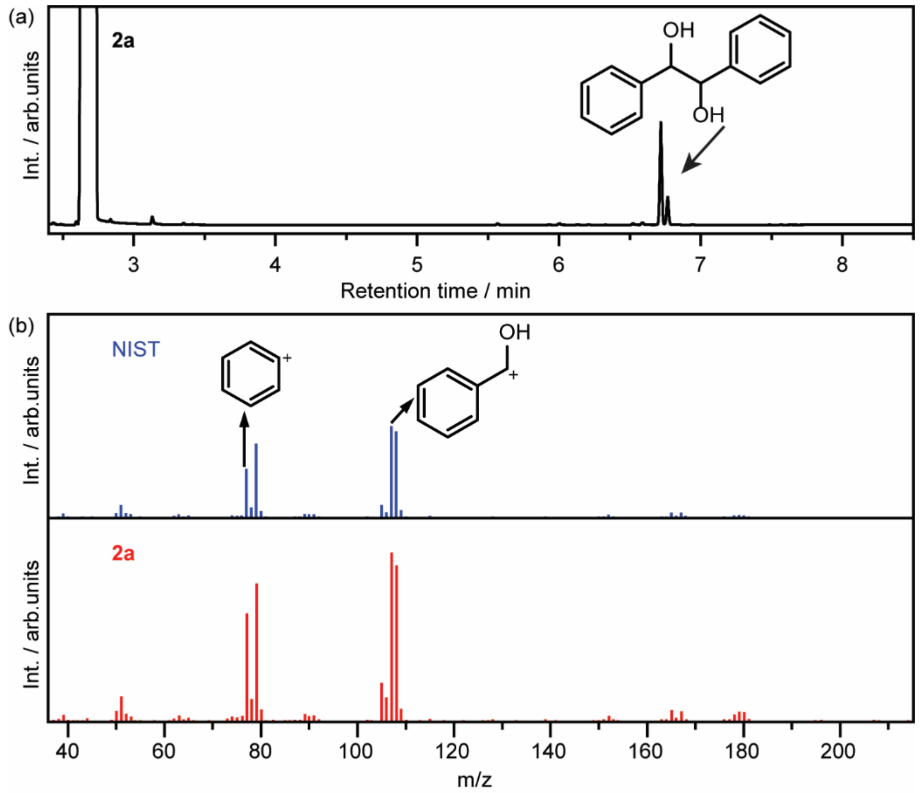

Figure S34. 1,2-diphenylethane-1,2-diol **2a**: (a) GC spectrum. (b) MS spectrum from GC-MS (above) and the standard MS spectrum from the NIST database (below).

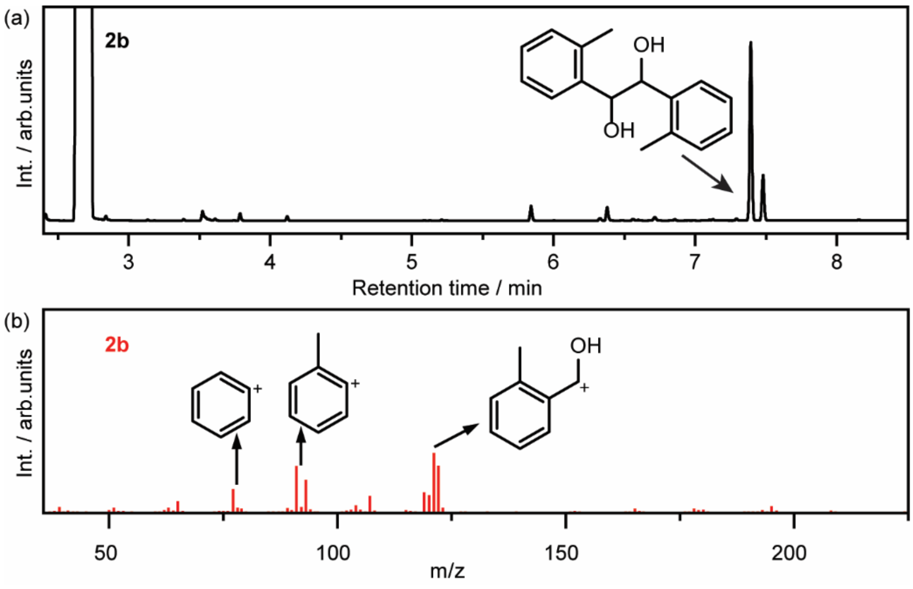

Figure S35. 1,2-di-*o*-tolylethane-1,2-diol **2b**: (a) GC spectrum. (b) MS spectrum from GC-MS.

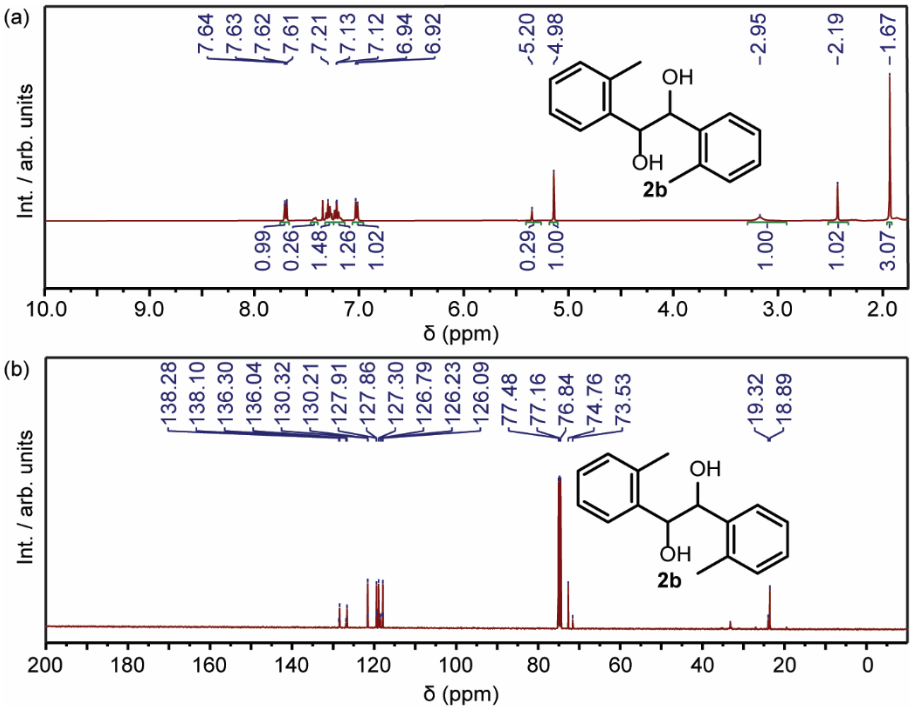

Figure S36. NMR spectra of **2b**. (a) ^{1}H NMR (400 MHz, $CDCl_3$) (peaks are reported for the mixture of both the *meso* and *dl* isomers): δ 7.63 (dd, *J* = 7.7, 1.5 Hz, 1H, *dl*), 7.37 – 7.31 (m, 3 × 1H, *meso*), 7.24 – 7.15 (m, 2H, *dl*), 7.12 (dd, *J* = 7.5, 1.4 Hz, 3 × 4H, *meso*), 6.93 (d, *J* = 7.5 Hz, 1H, *dl*), 5.20 (s, 3 × 1H, *meso*), 4.98 (s, 1H, *dl*), 2.95 (s, 3 × 1H, *dl*), 2.19 (s, 3 × 3H, *meso*), 1.67 (s, 3H, *dl*). (b) ^{13}C NMR (101 MHz, $CDCl_3$) δ 138.3, 138.1, 136.3, 136.0, 130.3, 130.2, 127.9, 127.9, 127.3, 126.8, 126.2, 126.1, 74.8, 73.5, 19.3, 18.9. The Spectral data for the product was in accordance with what was previously reported.[3]

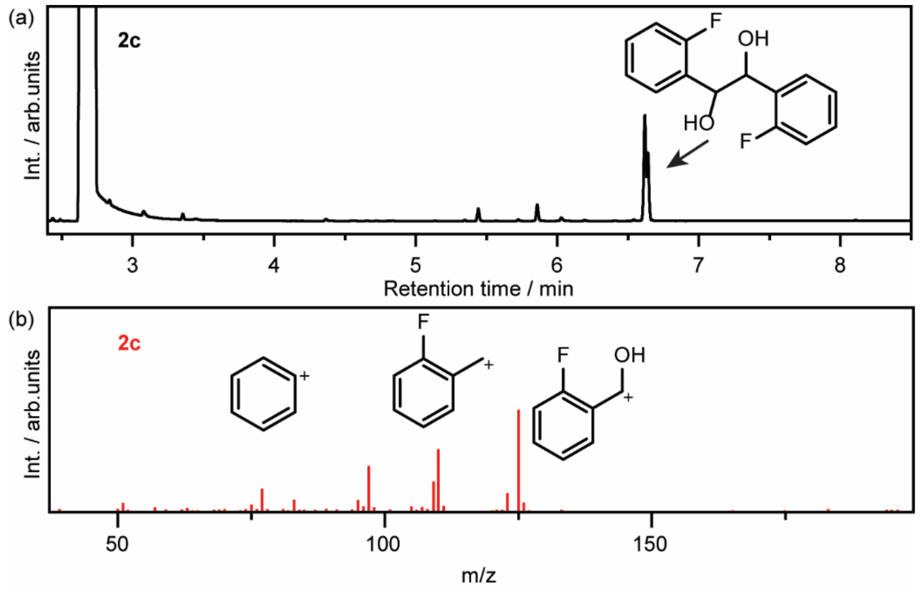

Figure S37. 1,2-bis(2-fluorophenyl)ethane-1,2-diol **2c**: (a) GC spectrum. (b) MS spectrum from GC-MS.

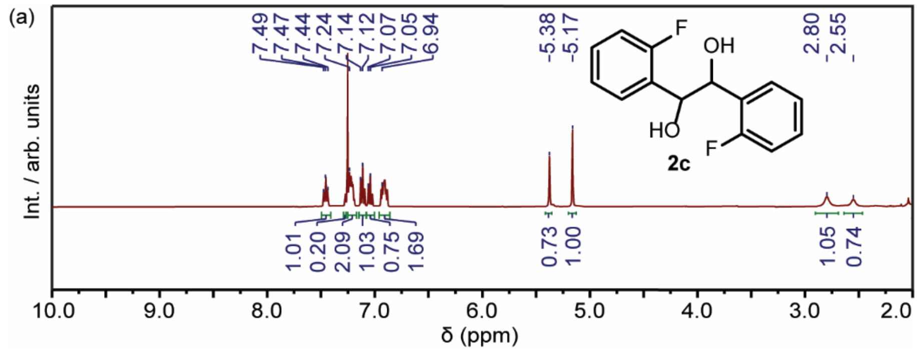

Figure S38. NMR spectra of **2c**. (a) ^{1}H NMR (400 MHz, $CDCl_3$) (peaks are reported for the mixture of both the *meso* and *dl* isomers): δ 7.46 (td, *J* = 7.4, 1.8 Hz, 1H), 7.24 (tdd, *J* = 10.8, 6.3, 2.4 Hz, 3H), 7.12 (td, *J* = 7.5, 1.2 Hz, 1H), 7.05 (td, *J* = 7.6, 1.1 Hz, 1H), 6.97 – 6.86 (m, 2H), 5.38 (s, 1H, *meso*), 5.17 (s, 1H, *dl*), 2.80 (s, 1H, *dl*), 2.55 (s, 1H, *meso*).

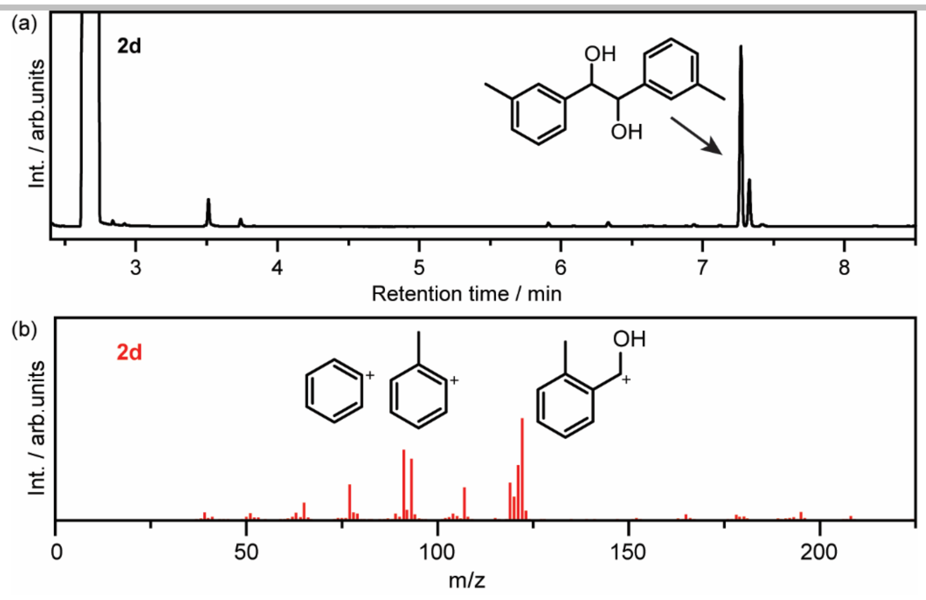

Figure S39. 1,2-di-*m*-tolylethane-1,2-diol **2d**: (a) GC spectrum. (b) MS spectrum from GC-MS.

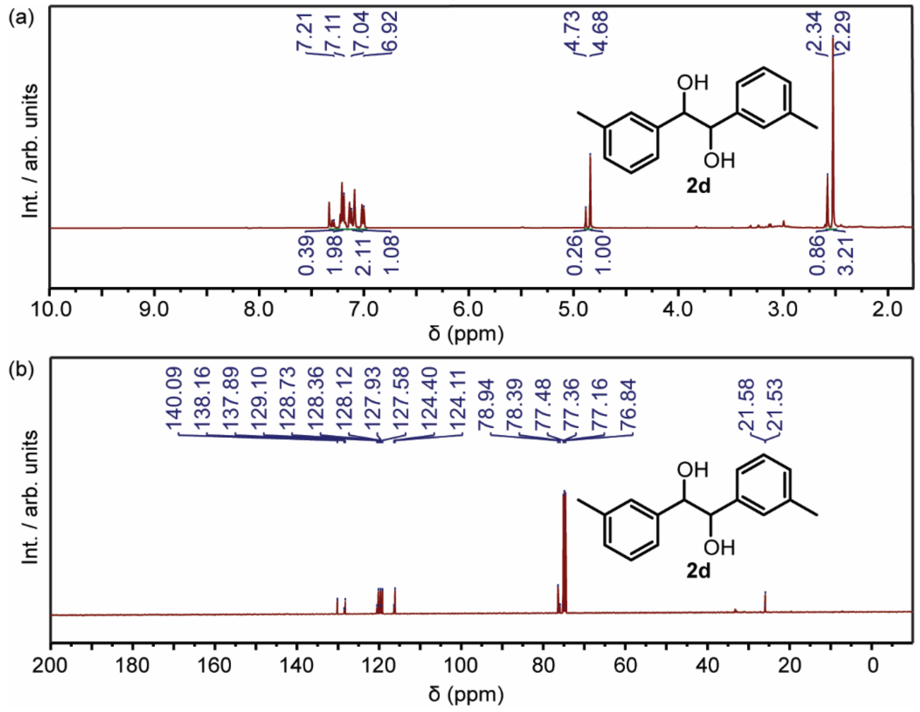

Figure S40. NMR spectra of **2d**. (a) ^{1}H NMR (400 MHz, $CDCl_3$) (peaks are reported for the mixture of both the *meso* and *dl* isomers):δ 7.23 (t, *J* = 7.5 Hz, 1H), 7.13 (t, *J* = 7.5 Hz, 3H), 7.08 – 6.99 (m, 3H), 6.93 (d, *J* = 7.5 Hz, 1H), 4.73 (s, 1H, *meso*), 4.68 (s, 1H, *dl*), 2.34 (s, 1H, *meso*), 2.29 (s, 3H, *dl*). (b) ^{13}C NMR (101 MHz, $CDCl_3$) δ 140.1, 138.2, 137.9, 129.1, 128.7, 128.4, 128.1, 127.9, 127.6, 124.4, 124.1, 78.9, 78.4.

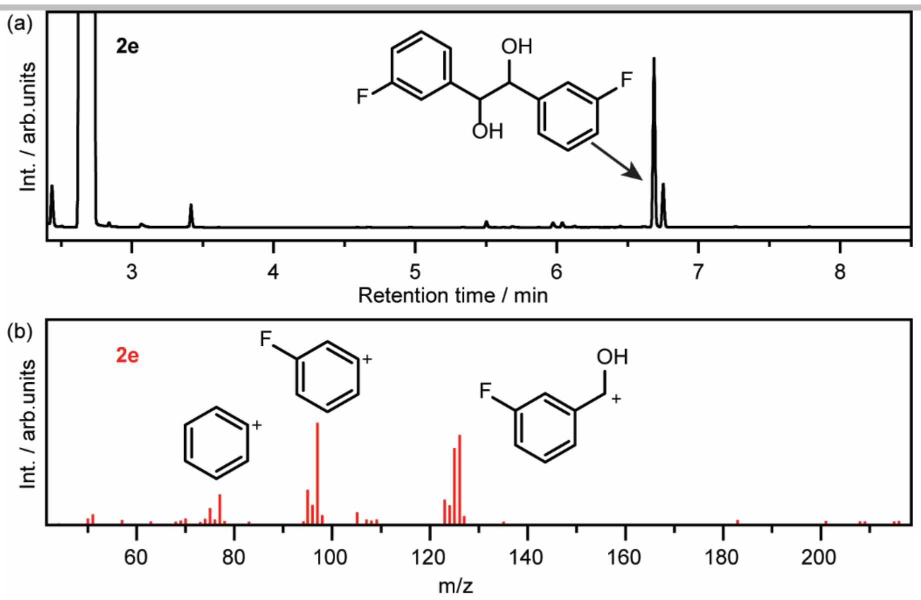

Figure S41. 1,2-bis(3-fluorophenyl)ethane-1,2-diol **2e**: (a) GC spectrum. (b) MS spectrum from GC-MS.

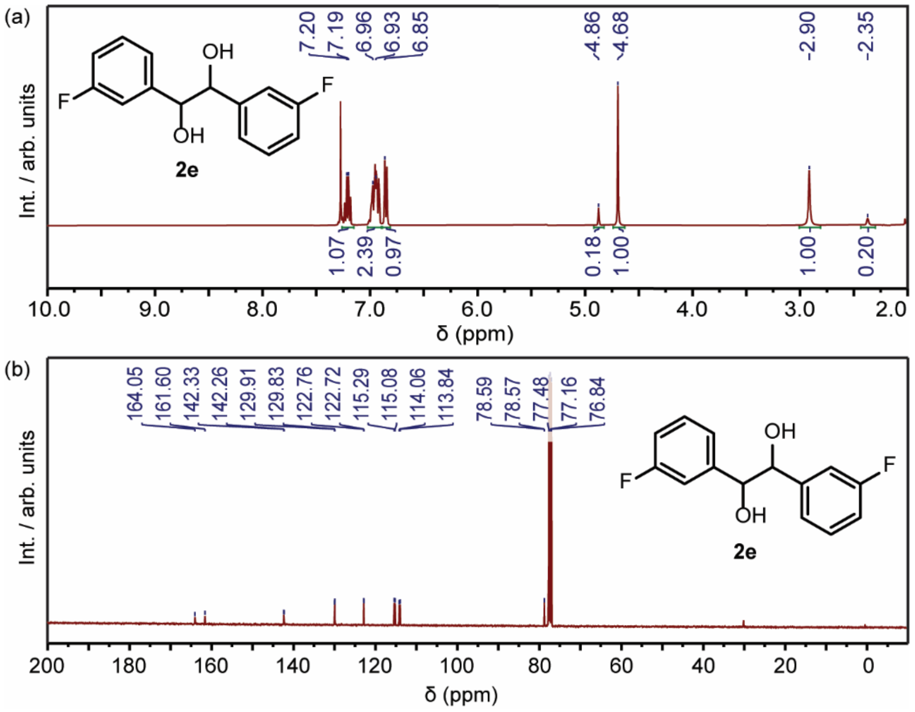

Figure S42. NMR spectra of **2e**. (a) ^{1}H NMR (400 MHz, $CDCl_3$) (peaks are reported for the mixture of both the *meso* and *dl* isomers): δ 7.19 (td, *J* = 7.9, 5.8 Hz, 2H), 7.01 – 6.88 (m, 5H), 6.84 (d, *J* = 7.7 Hz, 1H), 4.86 (s, 1H, *meso*), 4.68 (s, 1H, *dl*), 2.90 (s, 1H, *dl*), 2.35 (s, 1H, *meso*). (b) ^{13}C NMR (101 MHz, $CDCl_3$) δ 164.0, 161.6, 142.3, 142.3, 129.9, 129.8, 122.8, 122.7, 115.3, 115.1, 114.1, 113.8, 78.6, 78.6.

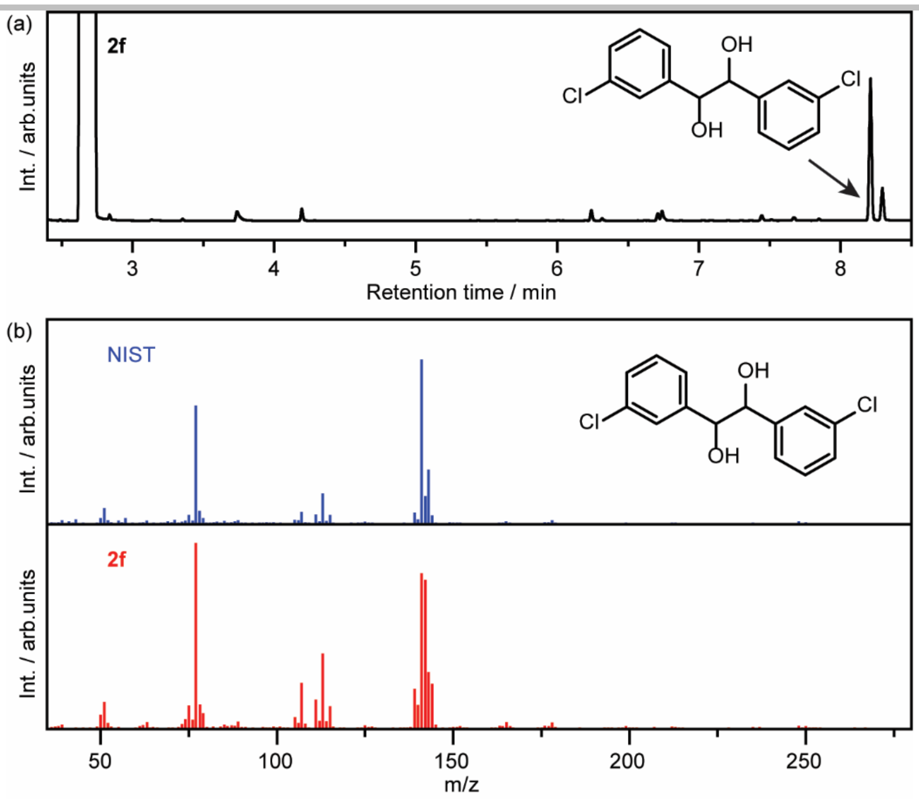

Figure S43. 1,2-bis(3-chlorophenyl)ethane-1,2-diol **2f**: (a) GC spectrum. (b) MS spectrum from GC-MS (above) and the standard MS spectrum from the NIST database (below).

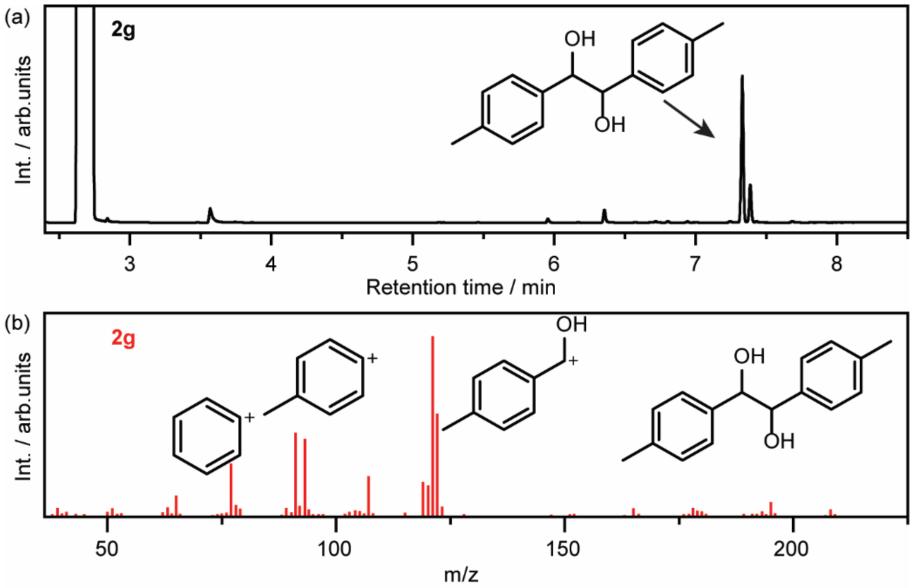

Figure S44. 1,2-di-*p*-tolylethane-1,2-diol **2g**:(a) GC spectrum. (b) MS spectrum from GC-MS.

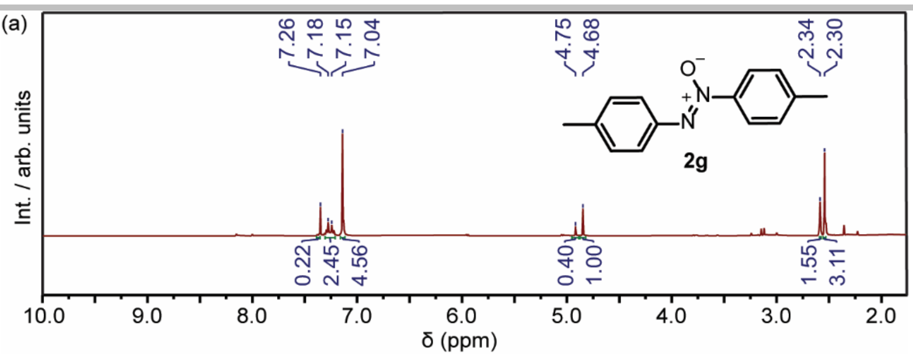

Figure S45. NMR spectra of **2g**. (a) ^{1}H NMR (400 MHz, $CDCl_3$) (peaks are reported for the mixture of both the *meso* and *dl* isomers): δ 7.23 – 7.09 (m, 3H), 7.04 (s, 5H), 4.75 (s, 1H, *meso*), 4.68 (s, 1H, *dl*), 2.34 (s, 3H, *meso*), 2.30 (s, 3H, *dl*). The spectroscopic data match the previously reported data.[14]

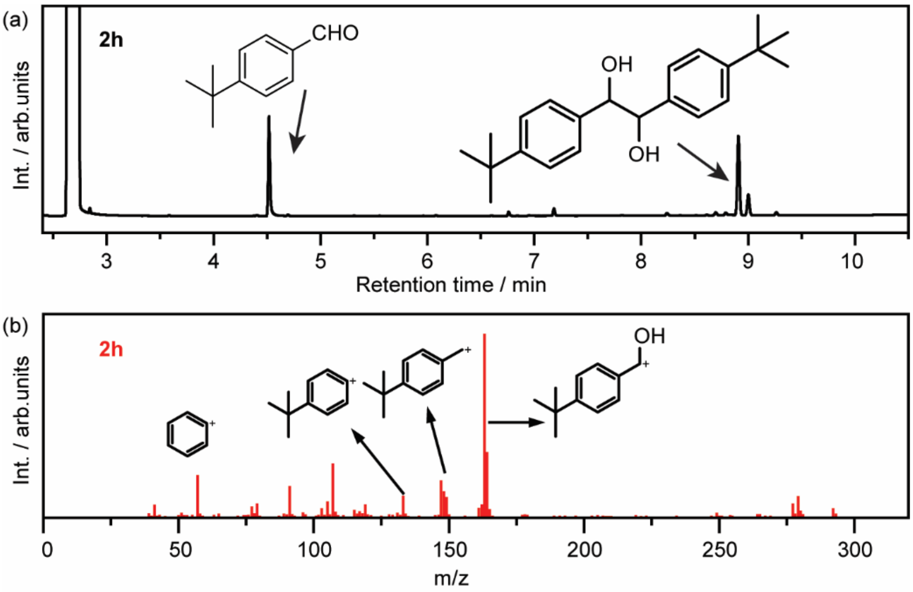

Figure S46. 1,2-bis(4-(tert-butyl)phenyl)ethane-1,2-diol **2h**: (a) GC spectrum. (b) MS spectrum from GC-MS.

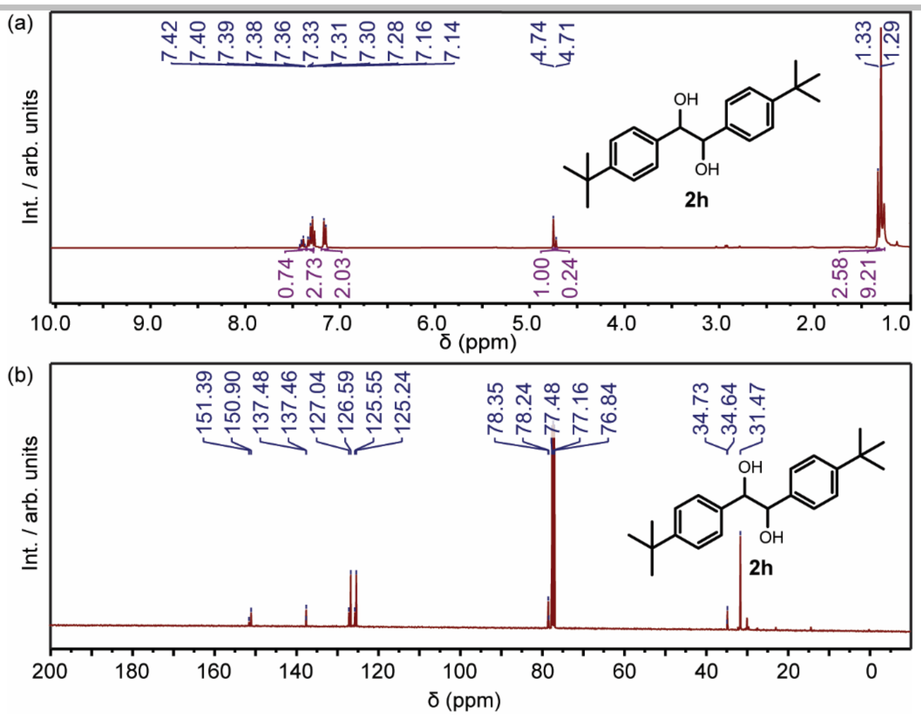

Figure S47. NMR spectra of **2h**. (a) ^{1}H NMR (400 MHz, $CDCl_3$) (peaks are reported for the mixture of both the *meso* and *dl* isomers): δ 7.39 (d, *J* = 8.1 Hz, 1H), 7.34 – 7.27 (m, 4H), 7.15 (d, *J* = 8.1 Hz, 3H), 4.74 (s, 1H, *meso*), 4.71 (s, 1H, *dl*), 1.33 (s, 9H, *dl*), 1.29 (s, 9H, *meso*). (b) ^{13}C NMR (101 MHz, $CDCl_3$) δ 146.6, 142.4, 140.7, 138.9, 137.4, 137.0, 129.9, 129.9, 127.0, 123.2, 123.1, 119.8, 20.1, 20.1, 20.0, 19.9. The spectroscopic data match the previously reported data.[3]

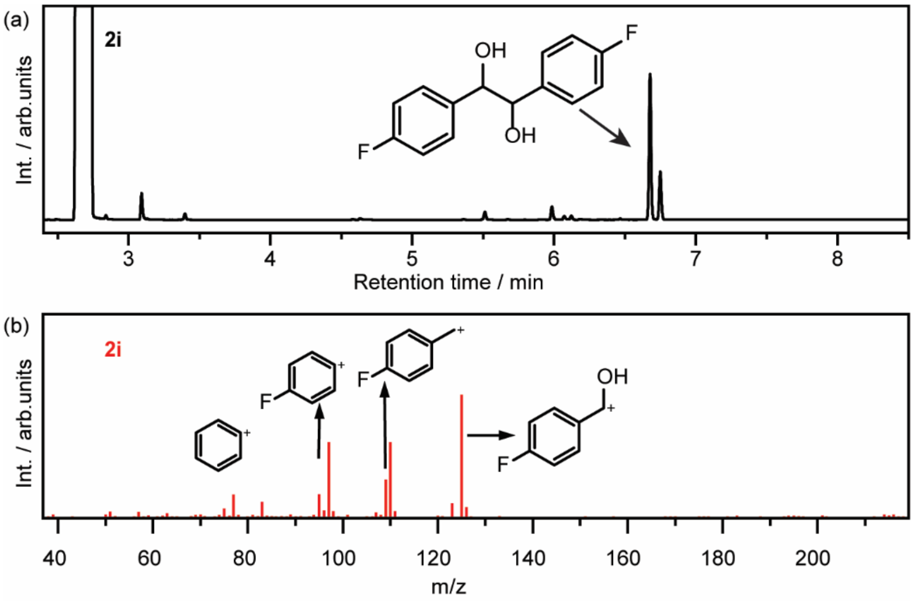

Figure S48. 1,2-bis(4-fluorophenyl)ethane-1,2-diol **2i**: (a) GC spectrum. (b) MS spectrum from GC-MS.

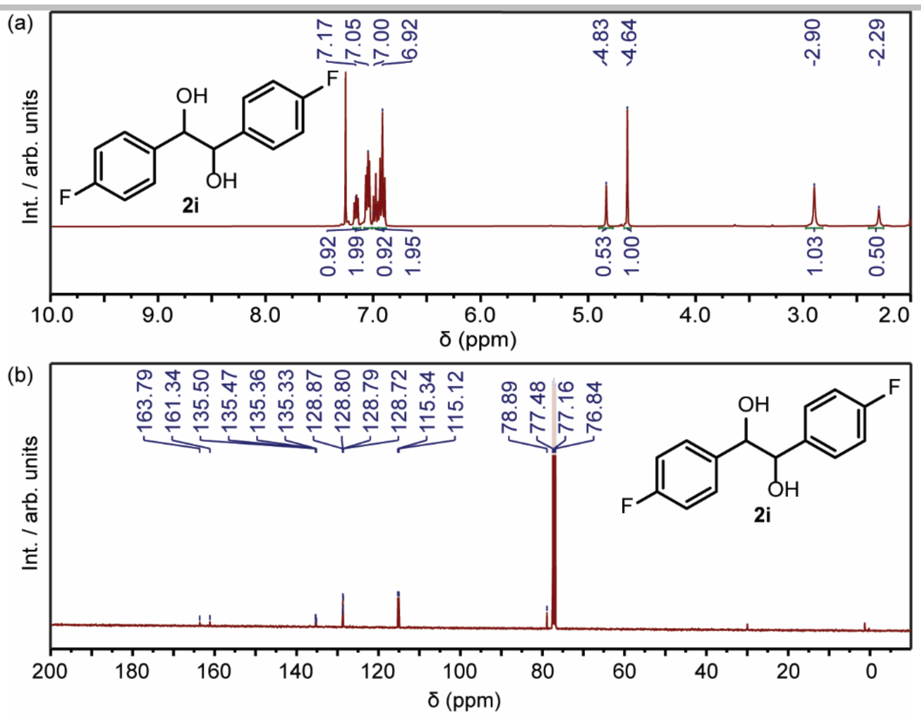

Figure S49. NMR spectra of **2i**. (a) ^{1}H NMR (400 MHz, $CDCl_3$) (peaks are reported for the mixture of both the *meso* and *dl* isomers): δ 7.20 – 7.12 (m, 1H), 7.11 – 7.02 (m, 3H), 7.01 – 6.95 (m, 2H), 6.95 – 6.88 (m, 2H), 4.83 (s, 1H, *meso*), 4.64 (s, 1H, *dl*), 2.90 (s, 1H, *dl*), 2.29 (s, 1H, *meso*). (b) ^{13}C NMR (101 MHz, $CDCl_3$) δ 163.8, 161.3, 135.5, 135.5, 135.4, 135.3, 128.9, 128.8, 128.8, 128.7, 115.3, 115.1, 78.9. The spectroscopic data match the previously reported data.[3]

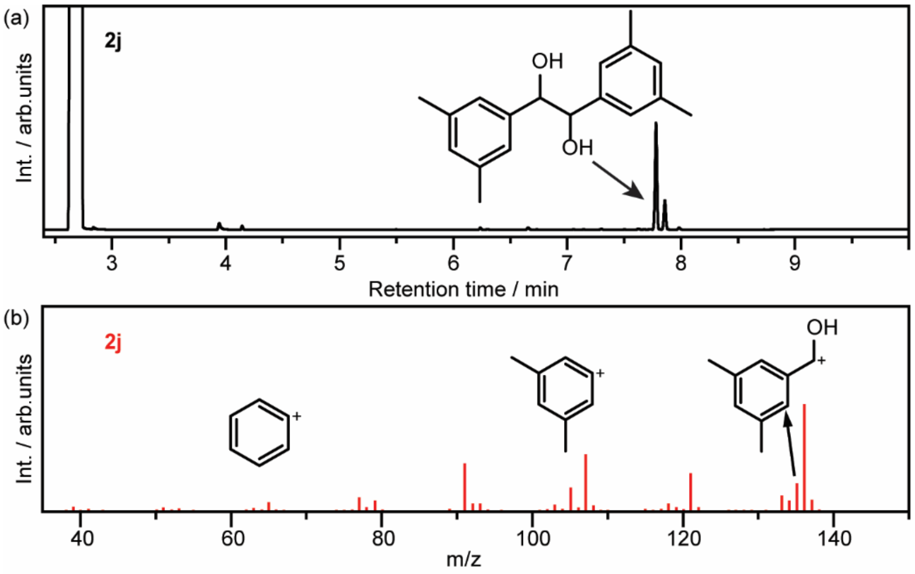

Figure S50. 1,2-bis(3,5-dimethylphenyl)ethane-1,2-diol **2j**: (a) GC spectrum. (b) MS spectrum from GC-MS.

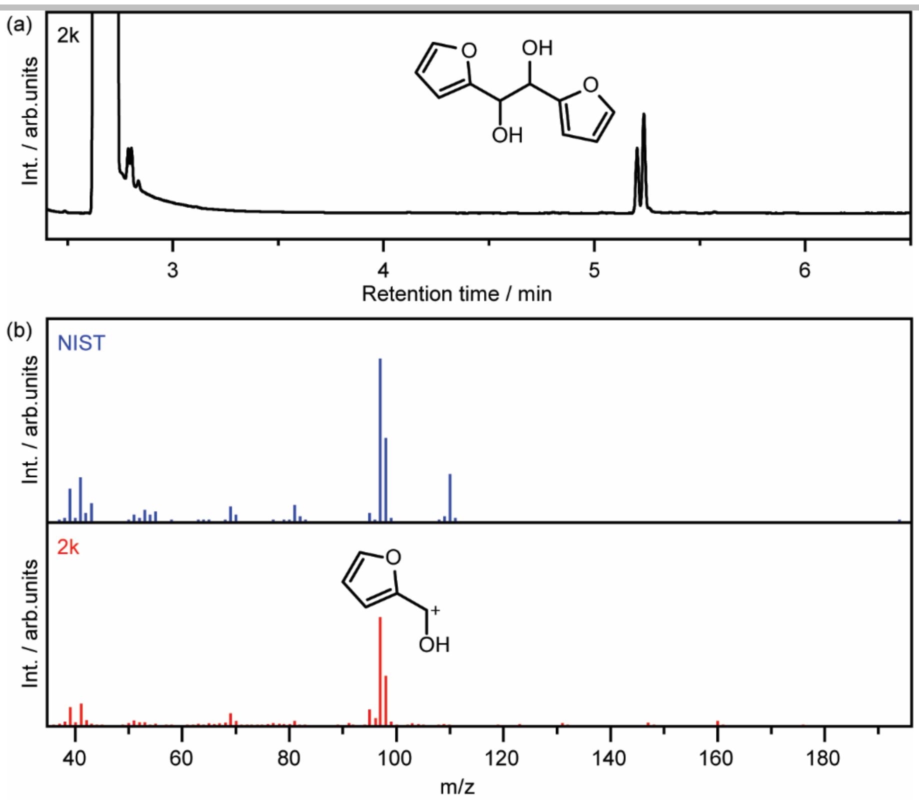

Figure S51. 1,2-di(furan-2-yl)ethane-1,2-diol **2k**: (a) GC spectrum. (b) MS spectrum from GC-MS (above) and the standard MS spectrum from the NIST database (below).

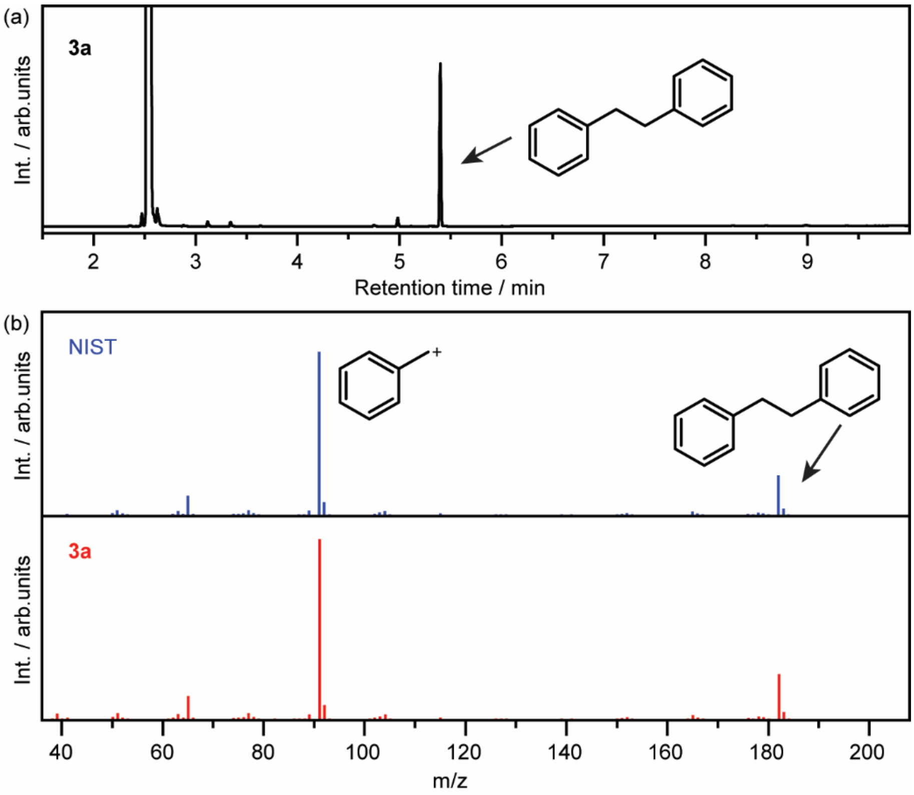

Figure S52. 1,2-diphenylethane **3a**: (a) GC spectrum. (b) MS spectrum from GC-MS (above) and the standard MS spectrum from the NIST database (below).

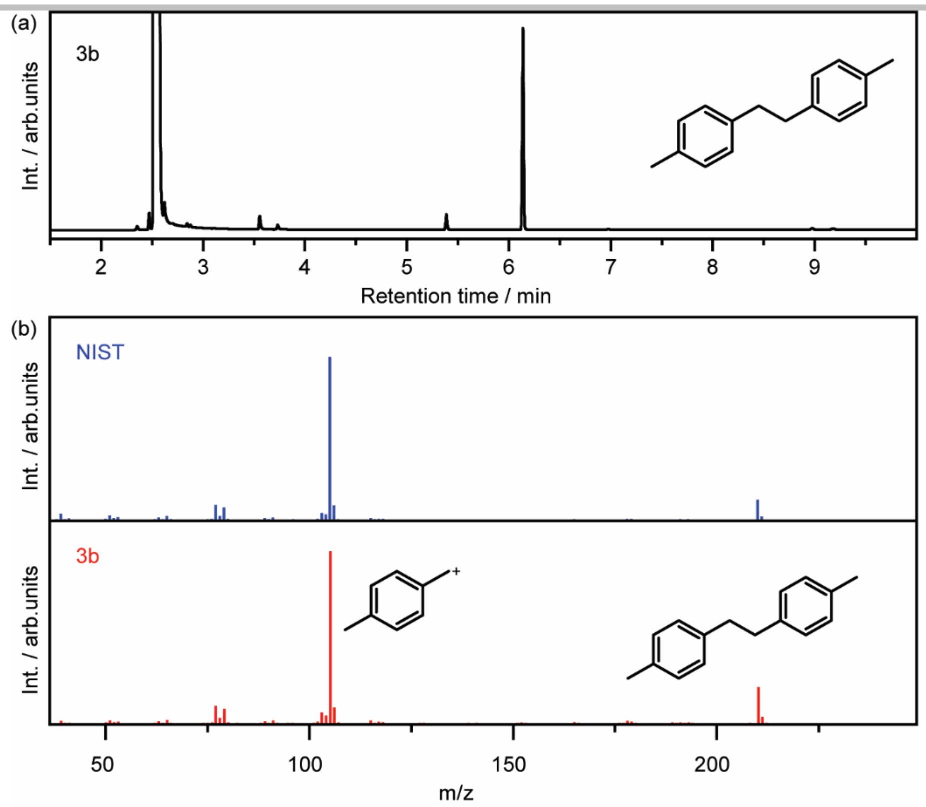

Figure S53. 1,2-di-*p*-tolylethane **3b**:(a) GC spectrum. (b) MS spectrum from GC-MS (above) and the standard MS spectrum from the NIST database (below).

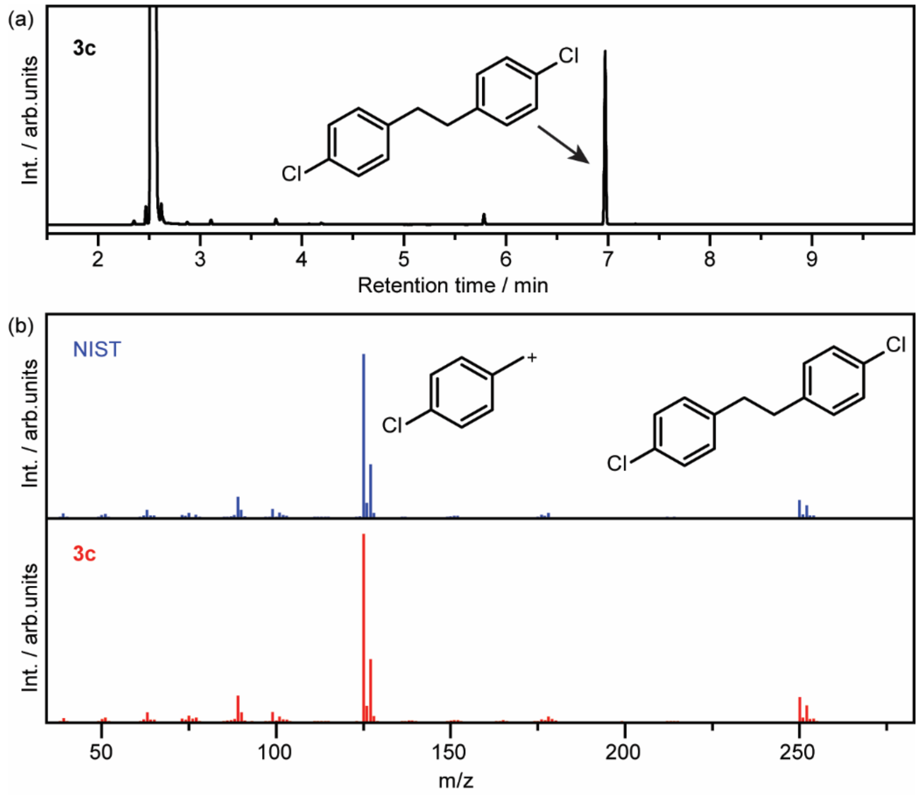

Figure S54. 1,2-bis(4-chlorophenyl)ethane **3c**: (a) GC spectrum. (b) MS spectrum from GC-MS (above) and the standard MS spectrum from the NIST database (below).

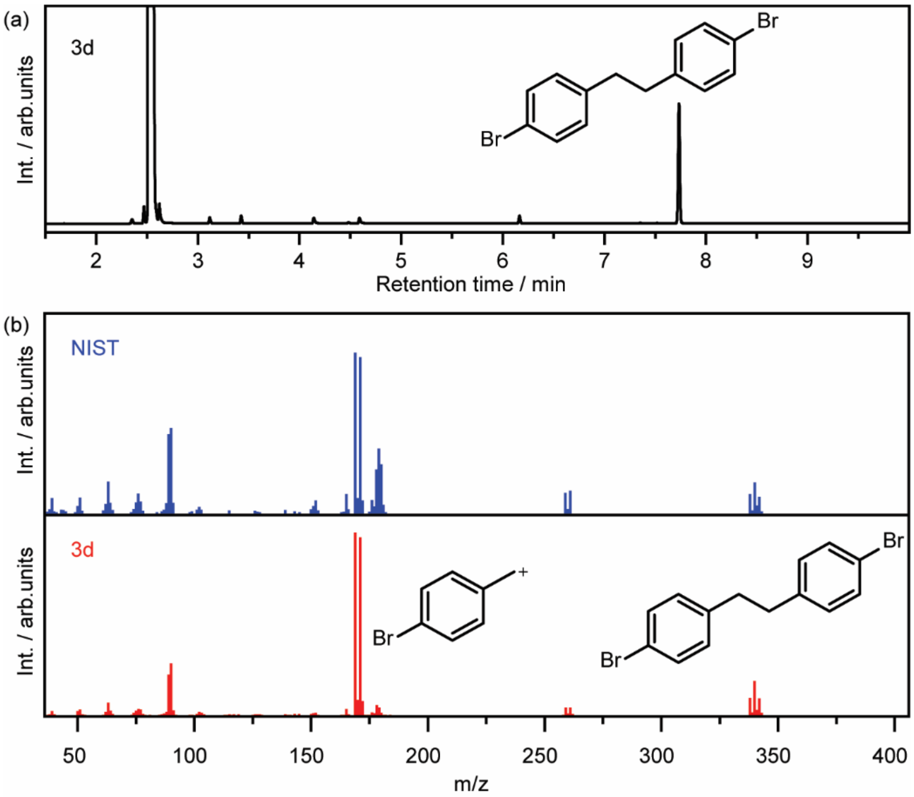

Figure S55. 1,2-bis(4-bromophenyl)ethane **3d**: (a) GC spectrum. (b) MS spectrum from GC-MS (above) and the standard MS spectrum from the NIST database (below).

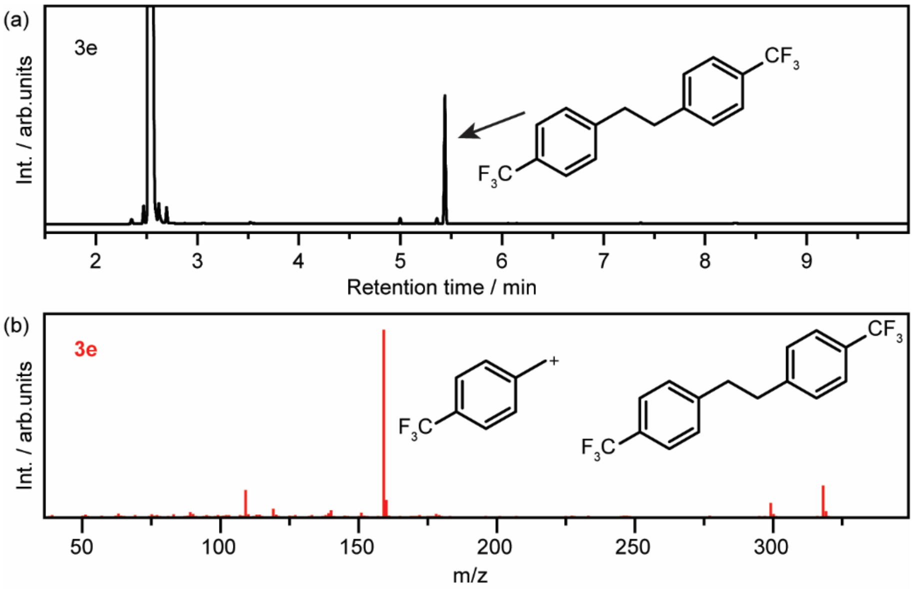

Figure S56. 1,2-bis(4-(trifluoromethyl)phenyl)ethane **3e**: (a) GC spectrum. (b) MS spectrum from GC-MS.

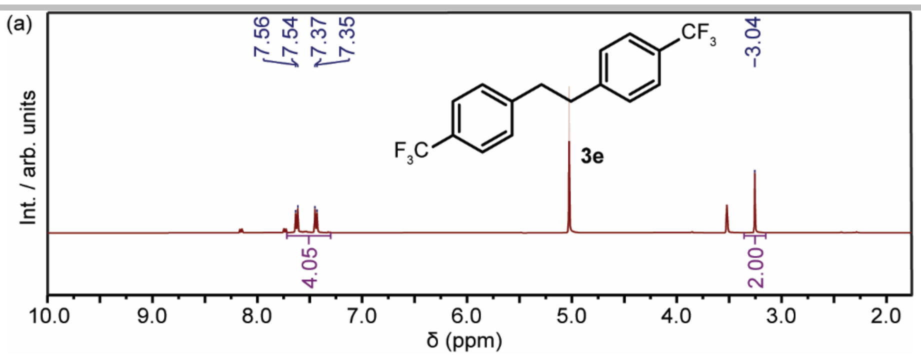

Figure S57. NMR spectra of **3e**. (a) ^{1}H NMR (400 MHz, MeOD) δ 7.45 (dd, J = 76.0, 7.9 Hz, 4H), 3.04 (s, 2H). The spectroscopic data match the previously reported data.[4]

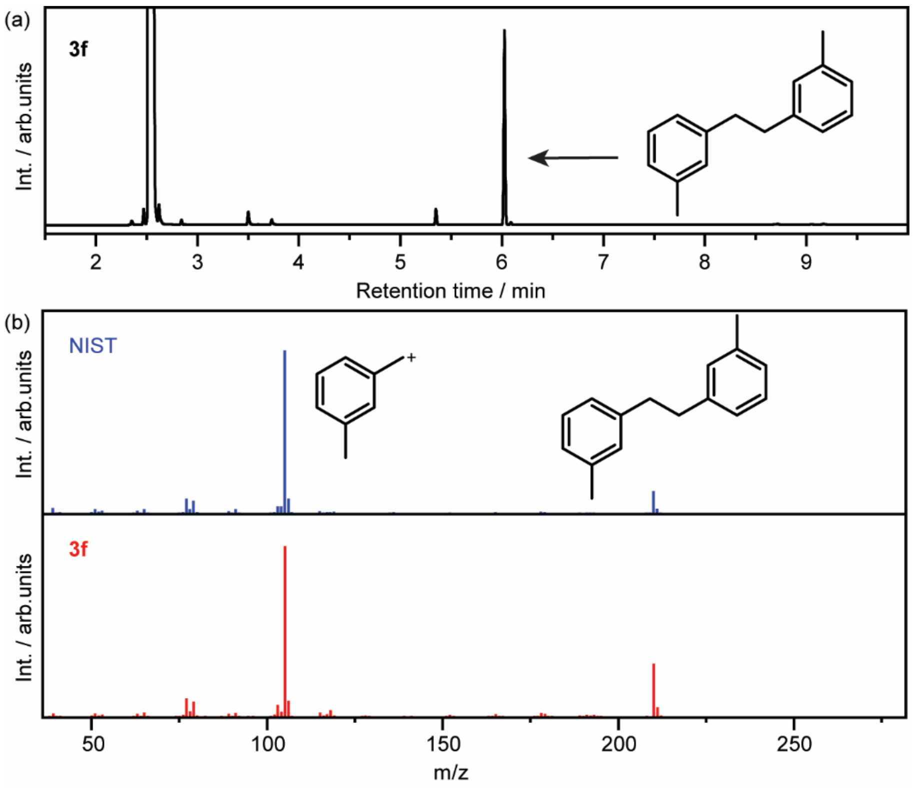

Figure S58. 1,2-di-*m*-tolylethane **3f**: (a) GC spectrum. (b) MS spectrum from GC-MS (above) and the standard MS spectrum from the NIST database (below).

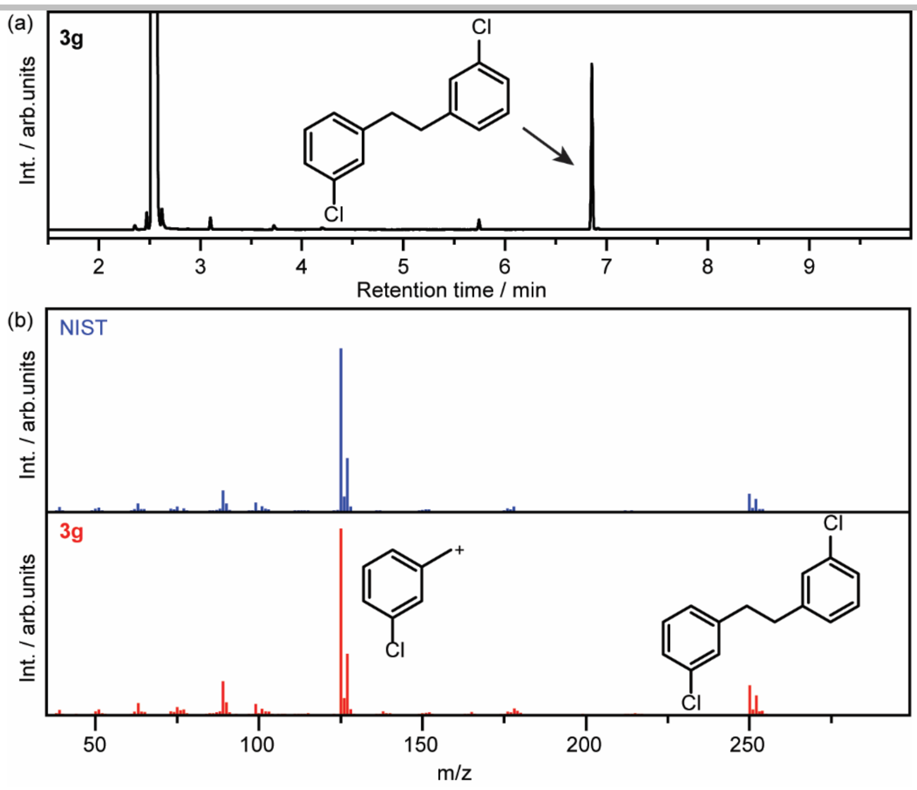

Figure S59. 1,2-bis(3-chlorophenyl)ethane **3g**: (a) GC spectrum. (b) MS spectrum from GC-MS (above) and the standard MS spectrum from the NIST database (below).

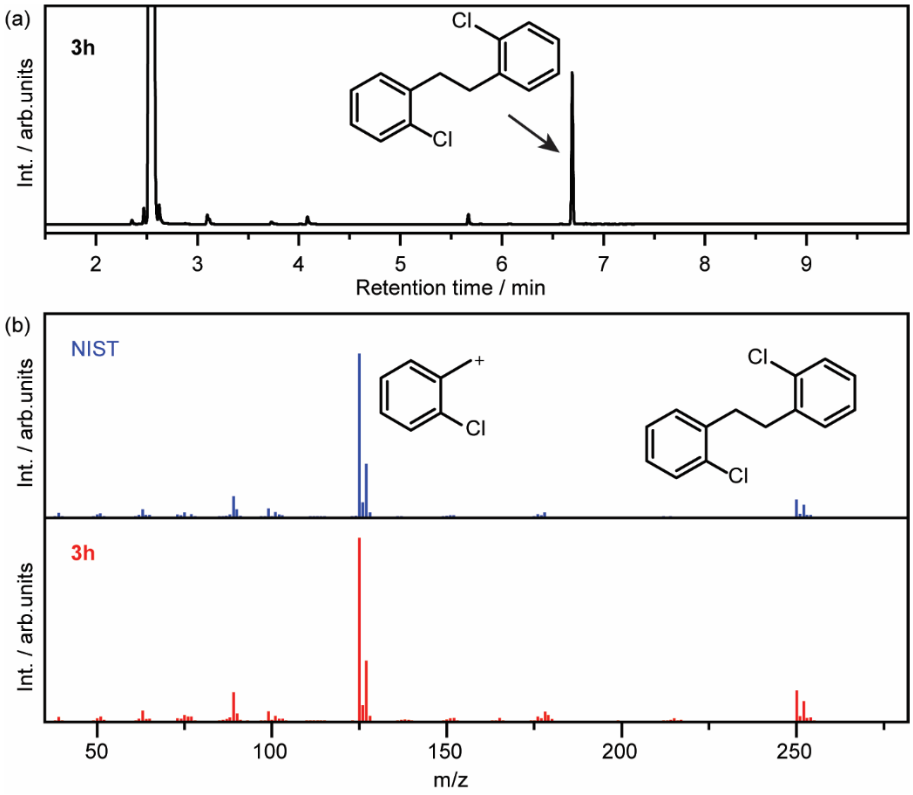

Figure S60. 1,2-bis(2-chlorophenyl)ethane **3h**: (a) GC spectrum. (b) MS spectrum from GC-MS (above) and the standard MS spectrum from the NIST database (below).

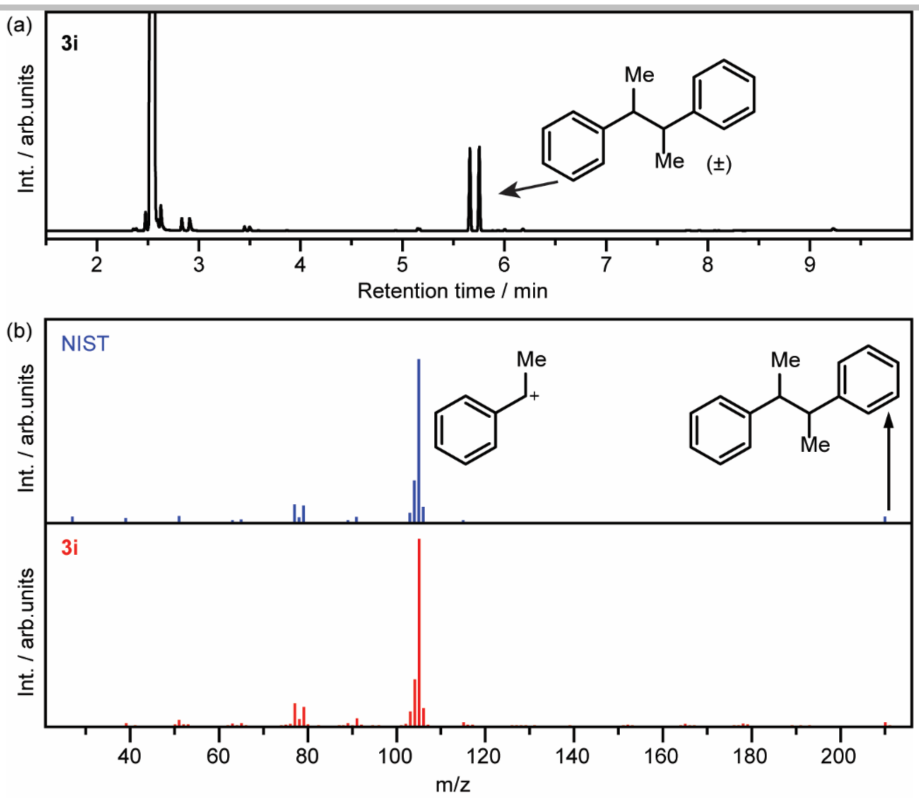

Figure S61. butane-2,3-diyldibenzene **3i**: (a) GC spectrum. (b) MS spectrum from GC-MS (above) and the standard MS spectrum from the NIST database (below).

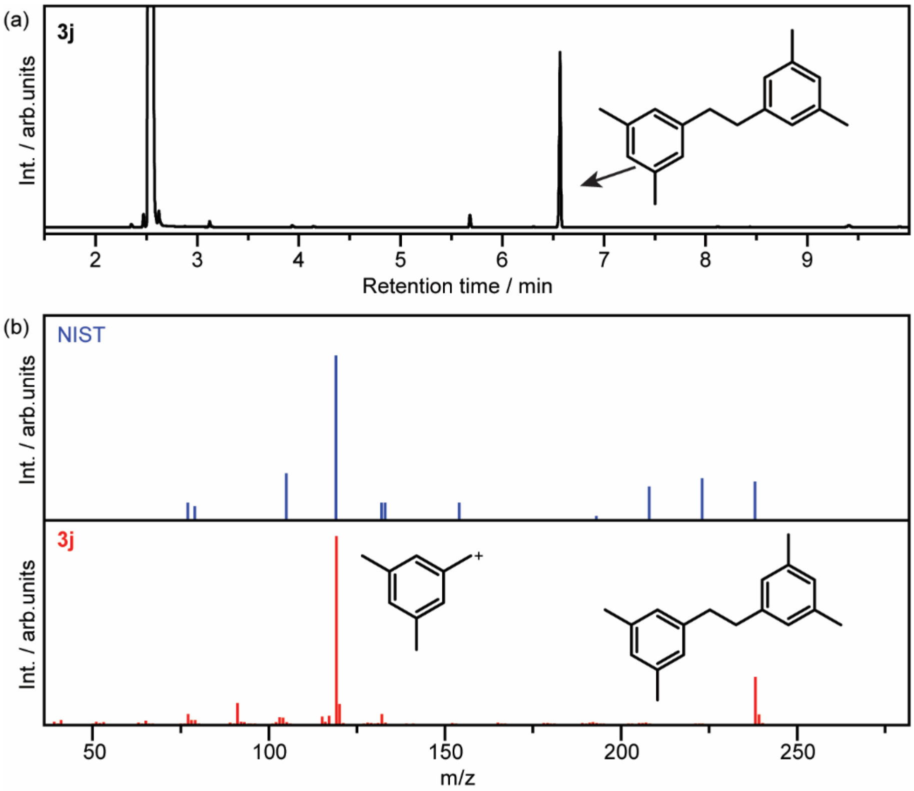

Figure S62. 1,2-bis(3,5-dimethylphenyl)ethane **3j**: (a) GC spectrum. (b) MS spectrum from GC-MS (above) and the standard MS spectrum from the NIST database (below).

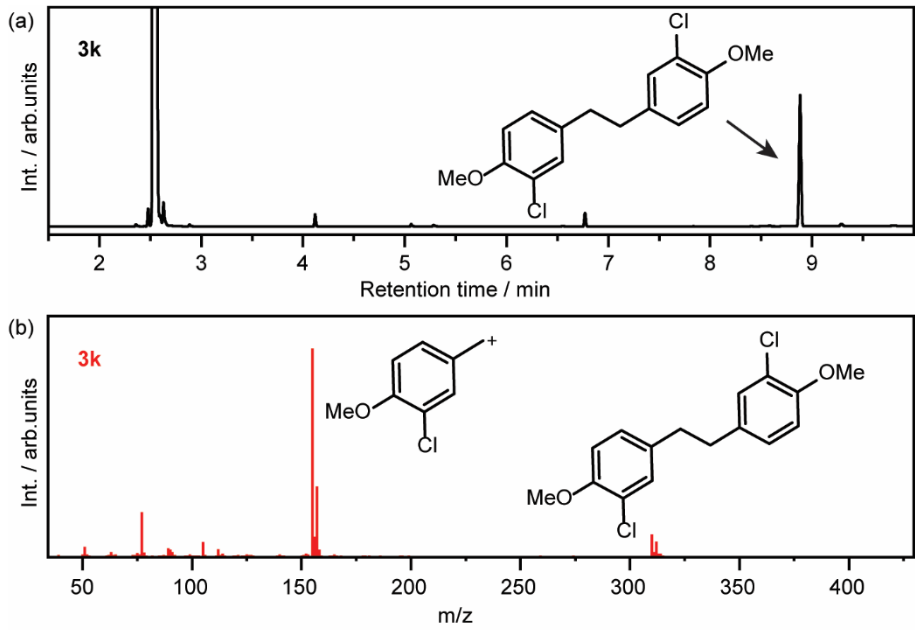

Figure S63. 1,2-bis(3-chloro-4-methoxyphenyl)ethane **3k**: (a) GC spectrum. (b) MS spectrum from GC-MS.

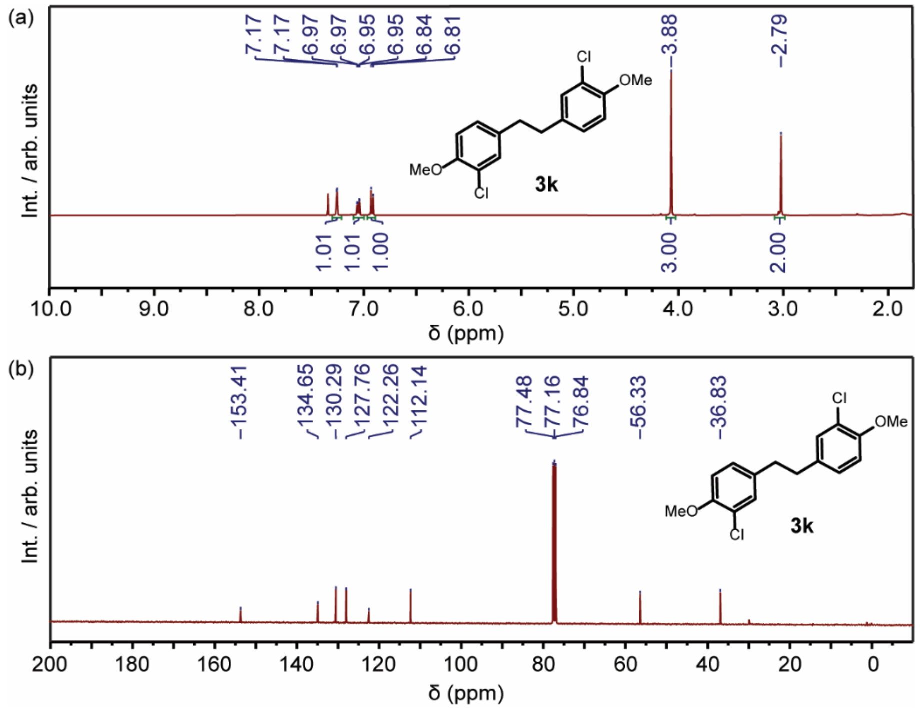

Figure S64. NMR spectra of **3k**. (a) ^{1}H NMR (400 MHz, $CDCl_3$) δ 7.17 (d, *J* = 2.2 Hz, 1H), 6.96 (dd, *J* = 8.4, 2.2 Hz, 1H), 6.82 (d, *J* = 8.4 Hz, 1H), 3.88 (s, 3H), 2.79 (s, 2H). (b) ^{13}C NMR (101 MHz, $CDCl_3$) δ 153.4, 134.6, 130.3, 127.8, 122.3, 112.1, 56.3, 36.8.

References

[1] X. Wang, K. Maeda, A. Thomas, K. Takanabe, G. Xin, J. M. Carlsson, K. Domen, M. Antonietti, *Nat. Mater.* **2009,** *8*, 76-80.
[2] Y. Dai, C. Li, Y. Shen, T. Lim, J. Xu, Y. Li, H. Niemantsverdriet, F. Besenbacher, N. Lock, R. Su, *Nat. Commun.* **2018,** *9*, 60.
[3] M. Nakajima, E. Fava, S. Loescher, Z. Jiang, M. Rueping, *Angew. Chem. Int. Ed.* **2015,** *54*, 8828-8832.
[4] Y. Li, P. Ren, D. Zhang, W. Qiao, D. Wang, X. Yang, X. Wen, M. H. Rummeli, H. Niemantsverdriet, J. P. Lewis, F. Besenbacher, H. Xiang, Y. Li, R. Su, *ACS Catal.* **2021,** *11*, 4338-4348.
[5] a) R. Ditchfield, *Mol. Phys.* **1974,** *27*, 789-807; b) J. R. Cheeseman, G. W. Trucks, T. A. Keith, M. J. Frisch, *J. Chem. Phys.* **1996,** *104*, 5497-5509.
[6] M. Frisch, G. Trucks, H. Schlegel, G. Scuseria, Robb, J. Cheeseman, G. Scalmani, V. Barone, B. Mennucci, G. A. Petersson. Gaussian 09W, revision A. 02. 2009.
[7] Y. Gao, G. Hu, J. Zhong, Z. Shi, Y. Zhu, D. S. Su, J. Wang, X. Bao, D. Ma, *Angew. Chem. Int. Ed.* **2013,** *52*, 2109-2113.
[8] a) V. Džimbeg-Malčić, Ž. Barbarić-Mikočević, K. Itrić, *Teh. Vjesn.* **2011,** *19*, 191-196; b) J. Tauc, R. Grigorovici, A. Vancu, *Phys. Status Solidi B* **1966,** *15*, 627-637.
[9] A. N. Baga, G. R. A. Johnson, N. B. Nazhat, R. A. Saadalla-Nazhat, *Anal. Chim. Acta* **1988,** *204*, 349-353.
[10] G. R. Buettner, *Free Radic. Biol. Med.* **1987,** *3*, 259-303.
[11] J. J. P. Stewart. MOPAC2016. Stewart Computational Chemistry, Colorado Springs, CO, USA, 2016.
[12] X. Chong, C. Liu, Y. Huang, C. Huang, B. Zhang, *Natl. Sci. Rev.* **2019,** *7*, 285-295.
[13] L. Chang, J. Li, N. Wu, X. Cheng, *Org. Biomol. Chem.* **2021,** *19*, 2468-2472.
[14] H. Kronenwetter, J. Husek, B. Etz, A. Jones, R. Manchanayakage, *Green Chem.* **2014,** *16*, 1489-1495.
